# Intrinsic stiffness and *Θ*-solvent regime in intrinsically disordered proteins: Implications for liquid–liquid phase separation

**DOI:** 10.1093/pnasnexus/pgaf039

**Published:** 2025-02-05

**Authors:** Lipika Baidya, Kurt Kremer, Govardhan Reddy

**Affiliations:** Solid State and Structural Chemistry Unit, Indian Institute of Science, Bengaluru, Karnataka 560012, India; Max Planck Institute for Polymer Research, Ackermannweg 10, Mainz 55128, Germany; Solid State and Structural Chemistry Unit, Indian Institute of Science, Bengaluru, Karnataka 560012, India

**Keywords:** finite-size effect, molecular dynamics simulations, condensate formation, coil-globule transition, amyloid formation

## Abstract

Liquid–liquid phase separation (LLPS) exhibited by intrinsically disordered proteins (IDPs) depends on the solvation state around the *Θ*-regime, which separates good from poor solvent. Experimentally, the *Θ*-solvent regime of the finite length (*N*) IDPs, as probed by small angle X-ray scattering (SAXS) and single molecular fluorescence resonance energy transfer (smFRET), is in disagreement. Using computer simulations of a coarse-grained IDP model, we address the effect of chain length on the *Θ*-regime of IDPs with polar side chains (polyglutamine) and hydrophobic side chains (polyleucine) subject to varying concentrations of cosolvents [C], urea (denaturant) or trimethylamine N-oxide (protective osmolyte) in water. Due to their intrinsic stiffness, these IDPs are always expanded on short-length scales, independent of the solvent quality. As a result, for short IDP sequences (≈1 to 3 residues), their propensity to exhibit LLPS cannot be inferred from single-chain properties. Further, for finite-size IDPs, the cosolvent concentration to attain the *Θ*-regime ($\left[C_{\Theta }\right]$) extracted from the structure factor emulating SAXS and pair distances mimicking smFRET differs. They converge to the same cosolvent concentration only at large *N*, indicating that finite size corrections vary for different IDP properties. We show that the radius of gyration ($R_{g}$) of the IDPs in the *Θ*-solvent regime satisfies the scaling relation $R_{g}^{2}=Nf(c\sqrt{N})$, which can be exploited to accurately extract $\left[C_{\Theta }\right]$ ($c=([C]/[C_{\Theta }]-1)$). We demonstrate the importance of finite size aspects originating from the chain stiffness and thermal blob size in analyzing IDP properties to identify the *Θ*-solvent regime.

**Significance Statement**

Intrinsically disordered proteins (IDPs) can cause pathological diseases through condensate formation. IDP propensity to form condensates correlates with its solvation state in the cell. In condensates IDPs mutually attract each other, similar to the precipitation of polymers out of solution. Experiments try to estimate this so called *Θ* condition for IDPs to predict their condensation. However, the published predictions for IDPs depend on the experimental technique and contribute to inconsistent conclusions. Here, we provide a universal scaling procedure that accurately estimates the *Θ* conditions for IDPs. We further show that shorter IDPs, due to their intrinsic stiffness, are expanded irrespective of solvent conditions, and the chain expansion is not a good descriptor for determining the IDP propensity to form condensates.

## Introduction

Intrinsically disordered proteins (IDPs) (1–3) are enriched with charged and polar residues and lack unique 3D structures. IDPs display rapidly interconverting conformations at physiological conditions. Despite or because of their disordered nature, IDPs are involved in many biological functions (2) related to stress granule assembly (4), cell signaling, chromatin remodeling, regulation of gene expression (5), signal transduction, etc. Additionally, IDPs also exhibit liquid–liquid phase separation (LLPS) and form biomolecular condensates (4, 6, 7) when present above the semidilute concentration and in poor solvent conditions. These biomolecular condensates in some cases further mature to form amyloid fibrils/aggregates (6, 8), which are implicated for neurodegenerative diseases. External factors such as salts (5, 9–12), pH (13–15), temperature (16), and cosolvents (17–20) alter the population of IDP conformations and LLPS propensity.

**Fig. 1.**
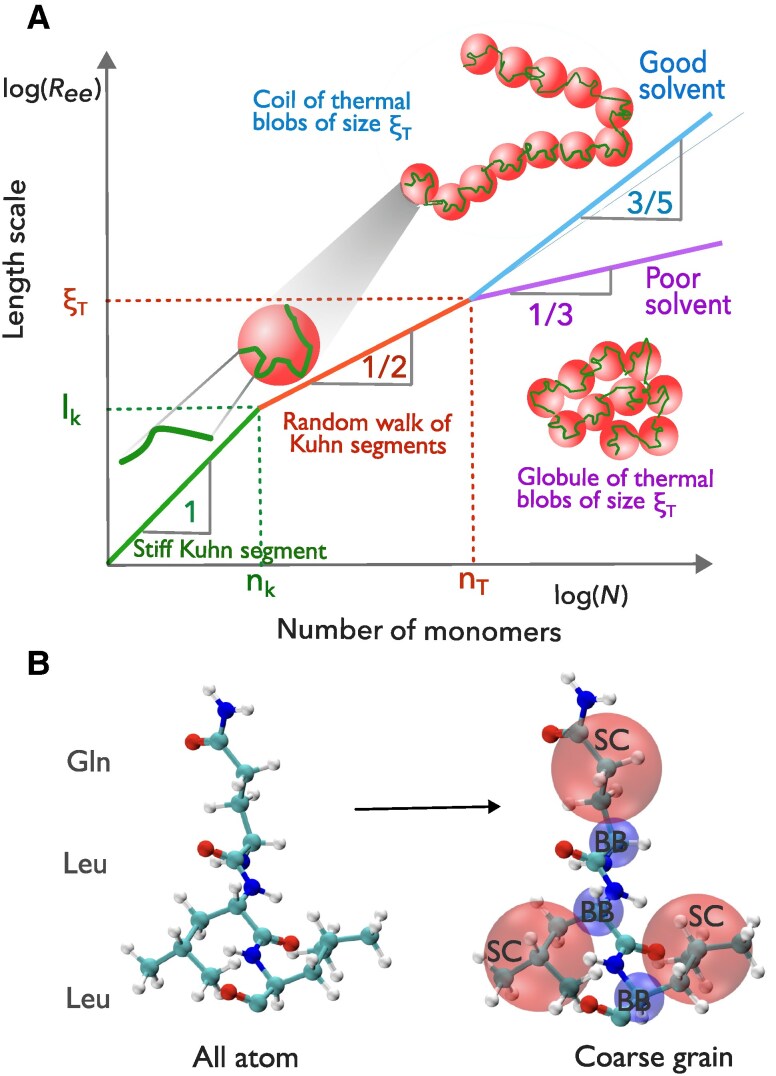
A) Blob Model: The size of the polymer chain is inferred using the end-to-end distance $R_{\mathrm{ee}}$ at different length scales as a function of the monomer number *N*. $l_{k}$ and $\xi_{T}$ are the Kuhn length and the thermal blob size, respectively. For $N>n_{T}$, the chain exhibits SAW of thermal blobs in good solvent and a collapsed globule of thermal blobs in poor solvent. B) SOP-IDP Model (Self-Organized Polymer Model for Intrinsically Disordered Proteins (21)): All-atom and coarse-grained representation of the tripeptide, Glutamine–Leucine–Leucine (Gln–Leu–Leu). The atomistic representation is shown on the left, where each atom in the amino acid residue is shown as a bead. The coarse-grained representation of the same tripeptide is shown on the right, where all the backbone (BB) atoms of an amino acid are represented as a single bead (blue), and the side chain (SC) atoms of the amino acid are represented by another bead (red).

**Fig. 2.**
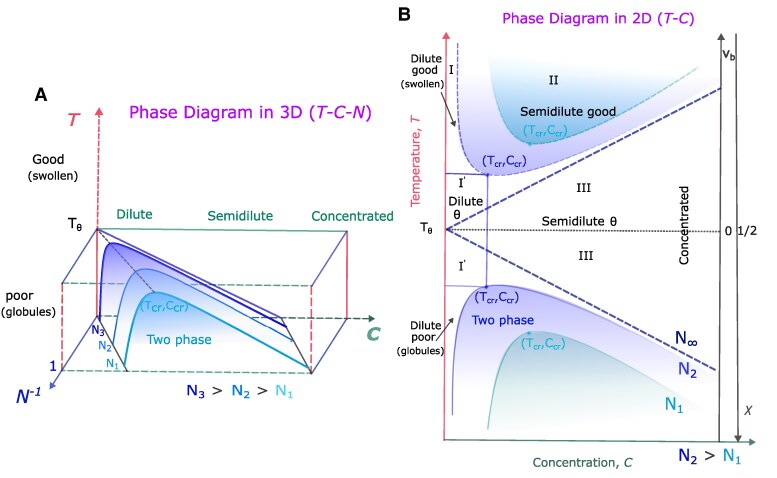
Schematic of the phase diagram of a polymer solution based on the Flory–Huggins theory. A) 3D phase diagram from a polymer-solvent system: The phase diagram is shown as a function of temperature (*T*), monomer concentration (*C*), and chain length (*N*) for three different chain lengths, $N_{1}$ (cyan), $N_{2}$ (light blue), and $N_{3}$ (dark blue), where $N_{3}>N_{2}>N_{1}$. Below the critical point ($C_{\mathrm{cr}}$, $T_{\mathrm{cr}}$) for a fixed *N*, the polymer phase separates into a polymer dilute phase and a dense phase. In the dilute phase, the polymer exists as globules and the dense phase is a polymer precipitate. The gray dashed line passes through the critical points ($C_{\mathrm{cr}}$, $T_{\mathrm{cr}}$) of the coexistence curves for all *N*. The theta temperature $T_{\Theta }$ is the tricritical point obtained when N→∞, and the low concentration segment of the coexistence curve merges with the *T* axis with a finite value for $T_{\Theta }$. B) Phase diagram of the polymer solution in two dimensions: The phase diagram is plotted as a function of *T* and *C* for two different chain lengths, $N_{1}$ (cyan) and $N_{2}$ (blue), where $N_{2}>N_{1}$. The good and poor solvent regimes are separated by a gray dotted line at $T_{\Theta }$. At $T_{\Theta }$, the excluded volume $\nu_{b}=0$ and Flory interaction parameter χ=1/2. Furthermore, $|T_{\mathrm{cr}}-T_{\Theta }|/T_{\Theta }∼n_{T}^{-1/2}∼1/\xi_{T}$, see part A). Regions I and II are the dilute and semidilute regions. Region III is the semidilute and concentrated region. Region ${\text{I}}^{\prime }$ is the dilute tricritical region, also known as the *Θ* region.

Although an IDP is a heteropolymer, insight into its phase separation propensity can be inferred from classical polymer physics scaling concepts, which form the basis of the generally accepted and experimentally proven blob model for polymers (22–24) (Fig. 1A). At short length scales, the polymer properties are determined by the local chemistry induced intrinsic stiffness of the chains. A measure of this length scale is the Kuhn length $l_{K}$ (which is twice the persistence length for a worm-like chain model, a special case of a freely rotating polymer chain model). Depending on the chemical details, $l_{K}$ typically corresponds to several monomers or amino acid residues. Any statistical description of the polymer or the IDP in terms of the blob model, strictly speaking, deals with length scales much larger than $l_{k}$. On length scales shorter than the Kuhn length segment ($l_{K}$), the polymer is stiff and independent of the solvent quality. The size of the Kuhn segment comprising of $n_{K}$ monomers scales as $l_{K}∼n_{K}$. On length scales between $l_{K}$ and the thermal blob size ($\xi_{T}$), the polymer behaves as a Gaussian chain as the excluded volume interactions are weaker than the thermal energy ($k_{B}T$). The size of the thermal blob scales as $\xi_{T}∼n_{T}^{1/2}$, where $n_{T}$ is the number of monomers comprising the thermal blob. On length scales larger than $\xi_{T}$, excluded volume repulsion energy exceeds $k_{B}T$ in good solvent conditions, and we observe a self avoiding random walk of thermal blobs. The size of the polymer scales as $R_{\mathrm{ee}}\approx \xi_{T}(N/n_{T}{)}^{3/5}$, where *N* is the number of monomers in the polymer. In poor solvent conditions, the excluded volume attraction energy exceeds $-k_{B}T$, and we observe a collapsed globule of thermal blobs and the size of the polymer scales as $R_{\mathrm{ee}}\approx \xi_{T}(N/n_{T}{)}^{1/3}$. The concentration dependent behavior of an IDP can be inferred from the phase diagram of a polymer solution (Fig. 2).

The schematic of a polymer solution phase diagram (22, 23) based on Flory–Huggins theory as a function of temperature (*T*), monomer concentration (*C*), and chain length (*N*) below the *Θ* temperature is shown in Fig. 2A. Finite-length polymers below their critical temperature ($T_{\mathrm{cr}}(N)$) and depending on their concentration, phase separate into a dilute solution of collapsed globules and a polymer-rich precipitate (Fig. 2A and B). $T_{\mathrm{cr}}(N)$ increases as $|T_{\mathrm{cr}}-T_{\Theta }|/T_{\Theta }∼1/\sqrt{N}$ and merges with $T_{\Theta }$, the *Θ* temperature, when N→∞. $T_{\Theta }$ is a tricritical point that separates the line of second-order transitions (“lambda”-transitions) from the first-order transitions (25–27). When N→∞, for $T<T_{\Theta }$, the curve-shaped liquid–liquid coexistence curve changes into an angle-like shape, and the two branches correspond to a pure solvent phase (C=0) and a phase containing polymer (Fig. 2A and B). Whereas for $T>T_{\Theta }$, a line of zero polymer concentration known as “lambda”-transitions corresponding to critical states characterized as self-avoiding walk singularities are observed. In the “lambda”-transitions in the polymer phase diagram, no real second-order phase transitions occur, but it is analogous to the “lambda”-transitions observed in other systems such as ${\mathrm{He}}^{3}$–${\mathrm{He}}^{4}$ showing tricritical points (27).

At the *Θ*-point, the polymer behaves as an ideal chain as the effective excluded volume $\nu_{b}=0$ (Fig. 2B). In dilute polymer solution, when $T>T_{\Theta }$, the solvent is known as a good solvent as $\nu_{b}>0$, and the polymer chain is swollen, and its size is greater than the ideal chain size. When $T<T_{\Theta }$, the solvent is poor as $\nu_{b}<0$, and the polymer collapses into a globule. For finite-length polymers, the transition between good and poor solvent conditions is not sharp, and there is a crossover region that scales as $|T_{\mathrm{cr}}-T_{\Theta }|/T_{\Theta }∼1/\sqrt{N}$. This crossover region is known as the *Θ*-regime, and the polymers exhibit ideal behavior in this regime (Fig. 2A and B). The solvent is poor when the temperature is below the upper critical solution temperature (UCST) $T_{\mathrm{cr}}$. Under such conditions, isolated polymers exist as collapsed globules in dilute solutions. The critical point below which LLPS occurs is at $T_{\mathrm{cr}}(N)$ and $C_{\mathrm{cr}}(N)$, the polymer overlap concentration known as the semidilute regime. Under these conditions, the chains undergo LLPS or precipitate into a polymer-rich and a dilute phase (Fig. 2A and B). In agreement with the polymer phase diagram (Fig. 2A and B), computer simulations of IDPs (28–33) showed that the UCST for LLPS ($T_{c}$) is highly correlated with the IDPs temperature to be in the *Θ*-regime at the single chain level. Thus, the IDP’s propensity to exhibit LLPS is encoded within its single-chain behavior. Therefore, to decode the LLPS propensity of IDPs, it is essential to understand the factors that influence the IDPs *Θ*-regime. We note that there are also systems (34, 35) which exhibit phase separation for temperatures above the lower critical solution temperature (LCST) as well, as observed in the upper half of the phase diagram (Fig. 2B).

In the vicinity of the *Θ* point, the correlation length $\xi_{T}$ to observe the Gaussian behavior scales as $\xi_{T}∼n_{T}^{1/2}∼(|T-T_{\Theta }|/T_{\Theta }{)}^{-1}$. For $N\gg n_{T}$, we observe poor or good solvent properties as illustrated in Figs. 1A and 2. Formally, this can be summarized in a crossover scaling scheme for the average chain end-to-end distance $\left\langle R_{\mathrm{ee}}^{2}(N)\right\rangle$ given by Refs. (22, 24, 36, 37)


(1) $$\langle R_{\mathrm{ee}}^{2}(N)\rangle^{1/2}∼N^{1/2}f_{\pm }(N\tau^{1/\phi }),$$

where $f_{\pm }(x)$ is a scaling function with $f_{\pm }(x)=\mathrm{const}$ for x→0 and $x^{\nu -1/2}$ for x→∞. The value of *ν* is the Flory exponent 3/5 and 1/3 in the good and poor solvent regimes, respectively, and *ϕ* is the tricritical crossover exponent (38). The most accurate value of the Flory exponent observed in good solvent conditions from simulations of extremely long chains is ν=0.5876 (39). This crossover scaling as described in Eq. 1 is well established for regular homopolymers. Although the function $f_{\pm }(x)$ for $R_{\mathrm{ee}}$, $R_{g}$, and $R_{h}$ are different, the scaling argument $N\tau^{1/\phi }$ as well as the asymptotic power laws are the same. However, finite size effects originating from finite *N* or small $n_{T}$ further away from the *Θ*-point can lead to deviations. In this article, we will use a similar scheme as a reference for interpreting the IDP simulation data obtained in different cosolvent concentrations. Temperature is widely used to modulate the properties of polymers. However, cosolvents such as urea and TMAO are used for IDPs because of their biological relevance. In this article, we propose a scaling argument to analyze different properties of finite-sized IDPs in the presence of the cosolvents.

The size of the IDPs is generally inferred using the radius of gyration ($R_{g}$), hydrodynamic radius ($R_{h}$), and end-to-end distance ($R_{\mathrm{ee}}$) (see Methods). These quantities are related to *N* through the same power law relation $R_{g}∼N^{\nu }$ or $R_{\mathrm{ee}}∼N^{\nu }$ or $R_{h}∼N^{\nu }$, where *ν* is the Flory scaling exponent (22, 23). However, in terms of the scaling scheme discussed above, different finite-size corrections towards the asymptotic power laws are expected (39, 40). If the IDP is in a good solvent, it exhibits a self-avoiding random walk (SAW) (ν=3/5), and if the IDP is in a poor solvent, it collapses to a globule (ν=1/3). The coil-to-globule (CG) transition occurs for long polymer chains (N→∞) when the solvent quality is changed from good through the *Θ*-point or the ideal state (ν=1/2) to the poor solvent regime. Previous studies on finite-length homopolymers have established that temperature-induced CG transition is not sharp and occurs through a crossover regime or *Θ*-regime (24). The width of the *Θ*-regime (24, 37) ($w_{\Theta }$) depends on *N* and varies as $w_{\Theta }∼N^{-1/2}$, cf. Fig. 2A and B. $w_{\Theta }$ vanishes at infinite chain length (N→∞). Theoretical and simulation studies (24, 37) have shown that the temperature for a polymer of length *N* to be in the *Θ*-regime ($T_{\Theta }^{N}$) follows the relation $T_{\Theta }^{N}-T_{\Theta }=mN^{-1/2}$.

Polymer theory is widely used in the interpretation of the IDP experimental data. Small-angle X-ray scattering (SAXS) experiments (41) on hydrophobic disordered proteins revealed that IDPs with high hydrophobicity and low net charge are expanded in water in contrast to the expected behavior of collapsed structures, which is observed in the FRET experiments (10, 42–44). From a polymer theory point of view, such deviations are not completely unexpected as different experimental techniques measure different moments of distances and the finite-size corrections depend on the property measured. Several studies contributed to understanding the source for the disagreement between the SAXS and FRET experiments in studying the collapse transition in proteins (41–51). As the IDPs studied are of not very large length, the chain length and the IDP composition can be crucial governing factors beyond temperature for the observed apparent CG transition. Hence, it is essential to understand the finite-size aspects of IDPs that can mislead in identifying the *Θ*-solvent regime and discover methods to accurately locate it from different IDP properties.

We studied the effect of chain length on the *Θ*-regime of the IDPs induced by cosolvents using computer simulations of coarse-grained models for hydrophilic polyglutamine (polyQ) and a hydrophobic polyleucine (polyL) by varying their chain lengths, *N*, from 16 to 512. In the coarse-grained model, each amino acid is represented by two beads, one for the backbone atoms and the other for the side chain atoms (Fig. 1B). We induce the CG transition in the IDPs using cosolvents urea and trimethylamine N-oxide (TMAO). Urea is a denaturant that destabilizes the compact state of IDPs. In contrast, TMAO is a protective osmolyte that stabilizes the compact state of IDPs. We used the molecular transfer model (MTM) (see the Supplementary material), which is an implicit solvent model, to study the effect of cosolvents on IDP properties. MTM has proven to be an excellent model to study the effect of cosolvents on biomolecules (52–54). Since MTM is an implicit solvent model, it cannot be used to study the co-nonsolvency or co-solvency effects, which might appear in mixed good solvents or poor solvents (55). This scenario is not relevant to the present study. We provide insights into the role of intrinsic stiffness of the IDP chains, which cannot be ignored in the case of short finite size IDPs, on the *Θ*-regime and contrast their behavior with long polymer chains. We further show that the estimated cosolvent concentration required for the IDPs to be in the *Θ*-regime ($\left[C_{\Theta }\right]$) depends on the IDP property measured and converges to the same concentration only in the case of long polymer chains. In addition, we provide a method to accurately estimate $\left[C_{\Theta }\right]$ and the IDP size in the *Θ*-regime.

## Results and discussions

### Size scaling of hydrophilic polyQ chains in water

**Fig. 3.**
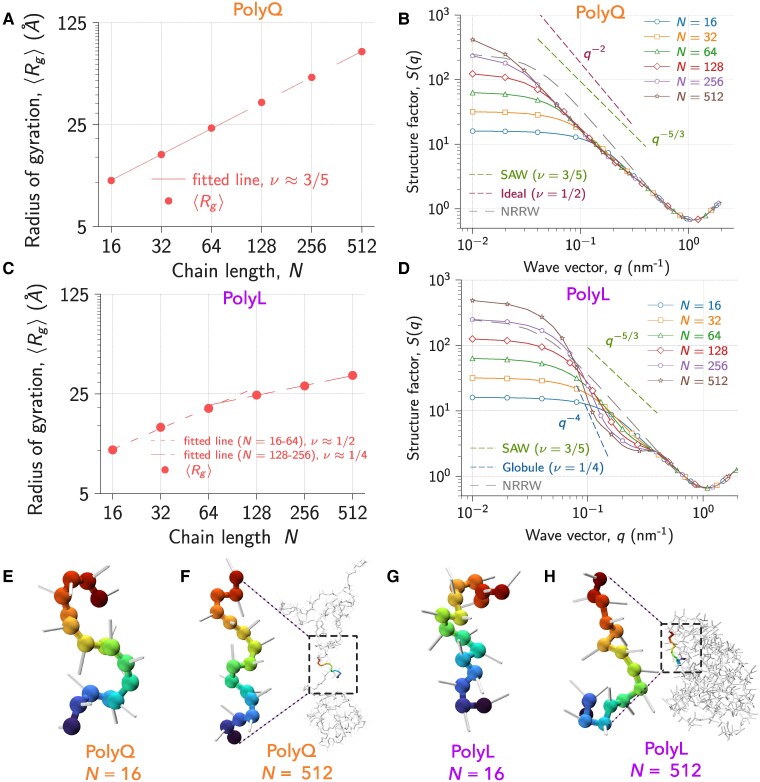
A) Scaling behavior of polyQ with *N* in water (no cosolvent). $\left\langle R_{g}\right\rangle$ plotted as a function of *N*. The fit of data (dashed line) to the equation $\log\;(\langle R_{g}\rangle )=\log\;(a)+\nu \log\;(N)$ yielded ν≈3/5. The error bars are on the same scale as the marker size. B) S(q) plotted as a function of *q* for polyQ chains for N=16–512. The gray dashed lines are the structure factor for nonreversal random walk (NRRW) for chain length, N=256. C) Scaling behavior of polyL with *N* in water. $\left\langle R_{g}\right\rangle$ plotted as a function of *N*. The fit of data (dashed line) to the equation $\log\;(\langle R_{g}\rangle )=\log\;(a)+\nu \log\;(N)$ yielded *ν* ≈ 1/2 for smaller polyL chains (N=16–64), and an apparent ν≈1/4 for longer chains (N=128–512). The error bars are on the same scale as the marker size. D) S(q) plotted as a function of *q* for polyL chains for N=16–512. The representative snapshots are shown for polyQ (E) N=16, (F) N=512, and polyL (G) N=16 and (H) N=512. The local stiffness for N=512 is shown by zooming a small segment of the IDP. The color gradient indicates residues from *N* (blue) to *C*-terminal (red).

We analyzed the conformational ensemble of polyQ chains of different lengths (N=16–512) in water (without the cosolvent) to probe the length dependence. The size of polyQ inferred using $\left\langle R_{g}\right\rangle$ ($\left\langle R_{h}\right\rangle$) increased from 10.35 (10.96) to 79.15 (58.44) Å for chain lengths ranging from N=16 to 512 (Fig. 3A and Supplementary material S1A). The monotonic increase in polyQ size as a function of *N* is in agreement with the fluorescence correlation spectroscopy (FCS) measurements (56) (Fig. S1). The $\left\langle R_{\mathrm{ee}}\right\rangle$ computed using simulations for shorter chain lengths (N≤30) is also in near quantitative agreement with the FRET (57) experiments (Fig. S1A and B). The scaling of protein size with *N* can be characterized using the Flory scaling exponent, *ν*. We fitted $\left\langle R_{g}\right\rangle$ using the equation, $\log\;(\langle R_{g}\rangle )=\log\;(a)+\nu \log\;(N)$ (where *a* is a constant) and recovered ν≈3/5, which denotes polyQ chains behave as a SAW (Fig. 3A). To assess the *ν* dependence on *N* for polyQ, we used multiple methods: (i) structure factor (S(q)), (ii) average pairwise interresidue distance ($\left\langle R_{\left|i-j\right|}\right\rangle$), and (iii) critical ratio ($\left\langle R_{\mathrm{ee}}^{2}\rangle /\langle R_{g}^{2}\right\rangle$).

We extracted *ν* from the structure factor, S(q) using the relation, $S(q)∼q^{-1/\nu }$, where *q* is the magnitude of the wave vector (Fig. 3B and Supplementary material S2A). For all the chain lengths (N=16–512), ν≈3/5, which indicates that the polyQ chains exhibit SAW (Fig. 3B and Supplementary material S2A). The SAW behavior also is evident from the Kratky plot (Fig. S2B), where the plots for all *N* are monotonically increasing. The critical ratio $\left\langle R_{\mathrm{ee}}^{2}\rangle /\langle R_{g}^{2}\right\rangle$ as shown in Fig. S3 displays an initial increase with *N* and then a leveling off within the error bars around the asymptotic value ($\langle R_{\mathrm{ee}}^{2}\rangle /\langle R_{g}^{2}\rangle \approx 6.25$) for SAWs (39, 40) for chains above a length of N=32. This is in agreement with the analysis of the scattering function above.

While the scattering function analyses internal distances in reciprocal space, the value of *ν* is also routinely extracted in real space from average interresidue distances ($\left\langle R_{\left|i-j\right|}\right\rangle$) using the relation $\langle R_{\left|i-j\right|}\rangle ∼|i-j{|}^{\nu }$, where *i* and *j* are residue indices (Fig. S2C). However, while computing $\left\langle R_{\left|i-j\right|}\right\rangle$ for chains of finite length, if *i* or *j* are close to the ends of the chain, it was shown that the distances between the points are distinctly smaller since the excluded volume repulsion is less important in such scenarios leading to significant errors in the interpretation of $\left\langle R_{\left|i-j\right|}\right\rangle$ (Figs. S4 and S5) (58). As a result, the extracted *ν* can be smaller, contributing to misleading conclusions about the state of the IDP. As |i−j| increases, the errors could be significant for finite length chains, mistakenly suggesting a crossover from SAW to Gaussian behavior (Fig. S2C). We also calculated $\left\langle R_{g}\right\rangle$ and $\left\langle R_{\mathrm{ee}}\right\rangle$ as a function of segment length *n* (see Methods) for polyQ chains, which yielded ν=3/5, similar to the calculations for S(q) and $R_{ij}$ (Fig. S6A,B).

### Comparison of computed PolyQ properties with experiments

We compared the $R_{h}$ and $R_{\mathrm{ee}}$ of polyQ computed using the simulation data from this work with the $R_{h}$ measured from the FCS experiments by Crick et al. (56) and $R_{\mathrm{ee}}$ measured from the FRET experiments by Walters and Murphy (57) (Fig. S1B). The lengths of the polyQ chains used in the FRET and FCS experiments are N≤30 and N≤60, respectively. The FRET efficiency (⟨E⟩) and $\left\langle R_{h}\right\rangle$ measurements confirmed that the size of polyQ increased monotonically with *N*. We observed that $R_{\mathrm{ee}}$ computed from simulations is larger than the FRET measurements, while the computed $R_{h}$ is 38% more compact than FCS measurements (Fig. S1B). The mismatch between simulations and experiments highlights the need to characterize the polyQ chain properties using longer chain lengths.

Previous molecular dynamics simulations using all-atom implicit solvent (59, 60) and explicit solvent models (61) indicated that polyQ chains in water exist in collapsed states and the size of polyQ chains varied with chain length as $N^{0.33}$. The collapse is due to the stronger intramolecular hydrogen bonds between the backbone secondary amide and the side chain amide groups (60, 62). Simulations using the coarse-grained model (Fig. 1B) in this work show that polyQ chains exhibit SAW behavior with increasing *N* for N≥32 (Fig. 3B). To probe the *N* dependent coil-globule transition, we calculated scaled $R_{g}$, $\langle R_{g}\rangle /\sqrt{N}$ as a function of *N* (Fig. S7A). We observed that $\langle R_{g}\rangle /\sqrt{N}$ monotonically increased with *N*, indicating a stable good solvent behavior of PolyQ with increasing *N* for chains up to N=512, significantly longer than those of previous studies. This conclusion is also supported by the Kratky plot of the scattering function (Fig. S2B). In neurodegenerative diseases involving glutamine residue, polyQ tracts of lengths greater than 30 are found to aggregate (63). The solubility of polyQ is also low in water, indicating it might be a poor solvent for polyQ (62, 64). The coarse-grained model used in this work might be overestimating the intrinsic stiffness in the polyQ chains, as discussed in the section below.

The primary goal of this work is to understand the properties of finite-length hydrophilic and hydrophobic-like polypeptide chains computed using multiple approaches mimicking various experimental techniques and the validity of polymer theory in analyzing these measurements and not to exclusively probe the single-chain properties of polyQ. Furthermore, it is important to clarify, which chain lengths are needed to infer solution behavior from single chain properties.

### Collapsed finite size hydrophobic polyL chains exhibit Porod scattering

To probe the effect of chain length on the conformations of hydrophobic polypeptides, we performed analogous simulations of polyL with different chain lengths (N=16–512) in water. The $\left\langle R_{g}\right\rangle$ ($\left\langle R_{h}\right\rangle$, $\left\langle R_{\mathrm{ee}}\right\rangle$) of polyL increased from 10.12 (10.85, 22.96) to 33.7 (34.38, 55.8) Å with *N* increasing from 16 to 512 (Fig. 3C and Supplementary material S8). For short chains (N=16) $R_{g}$ and $R_{h}$ of PolyQ and PolyL are essentially identical within the error bars, indicating that the intrinsic chain stiffness of the similar backbones dominates over the effective solvent induced interactions. However, compared to polyQ, the size of the polyL chains subsequently increased very slowly beyond N=16, as shown in Figs. S1A and S8. *ν* extracted by fitting $R_{g}$ to the equation $\log\;(\langle R_{g}\rangle )=\log\;(a)+\nu \log\;(N)$ revealed two different exponents, ν≈1/2 for shorter (N=16–64) and ν≈1/4 for longer (N=128–512) polyL chains (65) (Fig. 3C). The latter, of course, is an intermediate effective exponent possibly originating from different effects such as the collapse of a semiflexible chain and Porod scattering (see below), since an asymptotic exponent of 1/4 in three dimensions is impossible and *ν* eventually will be 1/3 for long chains. Thus polyL chains exhibit, just as polyQ chains, the crossover scaling proposed in Fig. 1A quite well.

To assess the finite size effects, we computed effective *ν* values using $\left\langle R_{\left|i-j\right|}\right\rangle$ for different *N* (Fig. S9C). *ν* computed using $\langle R_{\left|i-j\right|}\rangle ∼|i-j{|}^{\nu }$ gradually varied from ≈3/5 (SAW behavior) to a crossover value of 1/4 (dense compact globule will be 1/3) with increasing N=16 to 512 (Fig. S9C). This small *ν* intermediate apparent value of 1/4 is due to aggregation/folding of stiffer short segments, as shown in the conformation plots (Fig. 3H). The scattering function S(q) confirms a continuous change towards a compact globule, as also seen in the Kratky plot which changed from a monotonically increasing function with *q* for N≤32 to a nonmonotonic behavior for N≥64 indicating a transition from SAW-like structure to the globular state at N≈64 (Fig. S9B). Note that $S(q)∼q^{-1/\nu }$ only holds for fractals, i.e. self similar structures. A compact globule, however, is not a fractal structure and the power law $q^{-4}$ (Porod scattering (66)) originates from the sharp density step between the compact globule and the environment (see Supplementary material). In a perfect homogeneous globule, S(q) oscillates with periodicity π/R with an envelope scaling like $q^{-4}$. The exponent value between 1/3 and 1/4 indicates a rough interface. Scattering experiments (67) provide evidence that the globular proteins can exhibit the exponent 1/4. The bend in the $\left\langle R_{\left|i-j\right|}\right\rangle$ plot at |i−j|≈50 indicates a transition from an extended conformation to collapsed/globule states can occur for polyL chains with N>50 (Fig. S9C). The decrease in apparent *ν* with increasing polyL chain length is in agreement with the experiments (68). The critical ratio, $\left\langle R_{\mathrm{ee}}^{2}\rangle /\langle R_{g}^{2}\right\rangle$ exhibited a nonmonotonic behavior with *N* and the ratio is less than the Gaussian chain value (23) ($\langle R_{\mathrm{ee}}^{2}\rangle /\langle R_{g}^{2}\rangle =6$) for all *N* (Fig. S3). However for shorter chains (N≤32), the critical ratio appears to be close to the ideal chain value, and for the longer chains it decreased and approached the value for perfect globules (69) ($\langle R_{\mathrm{ee}}^{2}\rangle /\langle R_{g}^{2}\rangle \approx 3$) for chain length N=512. The scaled $R_{g}$, $\langle R_{g}\rangle /\sqrt{N}$ plotted as a function of *N* also showed a monotonic decrease for N≥64 suggesting that these longer chains prefer collapsed/globule states and the shorter chains (N≤32) exhibit extended Gaussian-like behavior (Fig. S7B) originating from short distance correlations along the backbone of the chains. Similarly, $\left\langle R_{g}\right\rangle$ and $\left\langle R_{\mathrm{ee}}\right\rangle$ as a function of segment length, *n* recover ν=1/2 at shorter length scale. However, at longer length scale, $\left\langle R_{g}\right\rangle$ and $\left\langle R_{\mathrm{ee}}\right\rangle$ vs. *n* recover ν=1/3 and 1/4, respectively (Fig. S6C and D).

### Intrinsic stiffness in finite length PolyQ and PolyL chains contributes to contrasting behavior compared to the long polymer chains

From the S(q) plot, we can understand the observation that depending on *N*, the polyL chains exhibit an expanded chain-like behavior similar to a SAW at short length scales (q≈0.4 to $1.0\;{\text{Å}}^{-1}$, i.e. approximately between 6 and 15 Å), and Gaussian to collapsed globule-like behavior at intermediate and large length scales (Fig. 3D). In poor solvents, polymers on length scales up to the thermal blob size exhibit Gaussian or ideal-chain behavior, and with the increase in chain length, a globule of thermal blobs is observed (Fig. 1A) (22, 23). However, the existence of a Gaussian thermal blob of size $n_{T}$ requires that it contains many segments of the Kuhn length $l_{k}$, i.e. $n_{T}\gg n_{k}$, where $n_{k}$ is the number of monomers comprising the Kuhn length $l_{k}$. Note that $l_{k}$ is essentially independent of the solvent quality. The crossover from this stiff regime leads to the observed SAW to Gaussian/globule-like behavior in short IDP chains with an increase in chain length (Fig. 3B and D) and should not be interpreted as a chain length dependent solvent quality.

On length scales q≈0.4 to $1.0\;{\text{Å}}^{-1}$, the scattering function S(q) for all *N* collapses onto a single curve, indicating that the solvent quality is the same for all chain lengths on this short length scale. (Note that for even shorter length scales, i.e. $q>1\;{\text{Å}}^{-1}$ the local bead packing along the chains dominates.) Here, PolyL exhibits an apparent SAW-like behavior (ν≈3/5) due to the intrinsic stiffness (Fig. 3D) and not due to solvent quality. To confirm this, we further performed simulations using the nonreversal random walk (NRRW) model (see Methods), which has local stiffness due to excluded volume interactions between residues on short-length scales and no interaction between residues on longer-length scales along the chain. On shorter length scales (q≈0.4 to $1.0\;{\text{Å}}^{-1}$), S(q) for the NRRW model collapsed onto the S(q) data of both polyL and polyQ chains for all *N*, and on longer length scales it deviates from the globule like scattering (Fig. 3D) for polyL, and SAW behavior (Fig. 3B) for polyQ, respectively.

The length of the intrinsic stiffness (l≈2π/q) extends to about 1 to 3 residues, assuming the size of glutamine and leucine residues are ≈6.0 Å (Table S2) (Fig. 3E–H). For polypeptides with sizes close to these length scales, we cannot infer the changes in solvent quality from the single chain properties and simulations with multiple chains are required to understand their aggregation propensity (28, 32). Experiments (70) have demonstrated that short peptides can undergo phase separation. To evaluate the LLPS propensity for short peptide chains whose size is on the length scale of their intrinsic stiffness, we need to perform multichain simulations, and cannot rely on identifying the *Θ*-regime from single chain properties.

### Chain length and residue type affect the IDP size in cosolvents

**Fig. 4.**
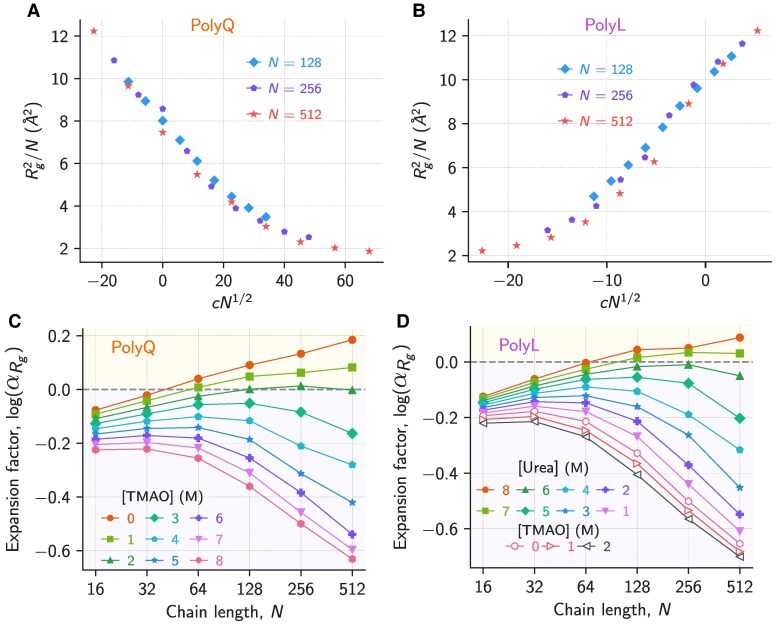
$R_{g}^{2}/N$ is plotted as a function of $cN^{1/2}$ for A) polyQ and B) polyL at various cosolvent concentrations, where $c=([C]/[C_{\Theta }]-1)$. For polyQ and polyL, the cosolvent concentration at *Θ* point, $C_{\Theta }$ are [TMAO]=2 M and [urea]=6.5 M, respectively. Expansion factor $\alpha_{R_{g}}$ is plotted as a function of *N* for C) polyQ and D) polyL at various solvent conditions. The solvent quality for polyL and polyQ is varied by increasing [TMAO] and [urea], respectively. $\alpha_{R_{g}}$ for each [C] is denoted with a different marker. The gray dashed line ($\log\;\alpha_{R_{g}}=0$) corresponds to the *Θ* condition. The yellow shaded region above the *Θ* point ($\log\;\alpha_{R_{g}}>0$) and the violet shaded region below the *Θ* point ($\log\;\alpha_{R_{g}}<0$) denotes good and poor solvent conditions, respectively.

The structure factor analysis showed that shorter polyQ and polyL chains exhibited similar SAW-like behavior in water due to local stiffness. The longer polyL chains formed globules (Fig. 3C and D and Supplementary material S9) and the polyQ chains (N≤512) exhibited SAW behavior (Fig. 3A and B and Supplementary material S2). We now modify the solvent quality to induce compaction or expansion of the IDP size to probe the CG transition (9, 41, 49) by altering the solvent conditions with the addition of cosolvents (10, 18, 19, 71) such as TMAO (protective osmolyte) and urea (denaturant), respectively. In the absence of cosolutes, polyQ chains exhibited SAW-like behavior for all *N* (Fig. 3). Hence, for polyQ, we used the protective cosolvent TMAO to induce the CG transition (Fig. 4). The behavior of polyL chains in the absence of cosolutes was dependent on *N*. The shorter polyL chains exhibited SAW-like behavior, whereas longer chains collapsed into globules (Fig. 3). Hence, to induce the CG transition in shorter and longer polyL chains, we used TMAO and urea, respectively (Fig. 4). We performed Langevin dynamics simulations in the presence of cosolvents (TMAO and urea) in the concentration range, [C]=1 to 8 M (see Methods and Supplementary material) to specifically probe the chain length effects on the CG transition induced by the cosolvents by studying the IDPs’ *Θ*-regime. The cosolvent is present implicitly in the model and is taken into account using the molecular transfer model (52–54).

We monitored the cosolvent (TMAO/urea) effect on polyL and polyQ by computing $\left\langle R_{g}\right\rangle$ as a function of cosolvent concentration [C] (Fig. S10). As TMAO stabilizes the compact states, $\left\langle R_{g}\right\rangle$ of polyQ and polyL decreased for all *N* with increasing [TMAO]. In contrast, urea stabilizes expanded IDP conformations, and the $\left\langle R_{g}\right\rangle$ of polyQ and polyL increased for all *N* with the increase in [urea]. The observed denaturing and stabilizing effect of urea and TMAO are consistent with previous experiments on IDPs (9, 19, 50). The variation in $\left\langle R_{g}\right\rangle$ for all *N* depended on the nature of the cosolvent and its concentration, and the extent of IDP expansion or compaction strongly depended on *N*. It should be noted that the effect of adding cosolvents here should not be confused with the co-nonsolvency effect, where the competition of two good solvents can lead to a chain collapse at intermediate concentrations of the added cosolvent (55).

We quantified the extent of expansion/compaction as a function of [C] by calculating the normalized radius of gyration, $\langle R_{g}\rangle /\sqrt{N}$ (Fig. S11). The extent of compaction in polyQ with increasing [TMAO] from 0 to 8 M is ≈16% for N=16 and ≈61% for N=512 indicating chain length dependent cosolvent induced compaction for IDPs. A similar extent of compaction is observed for polyL for chains of length N=16 (≈8%) and N=512 (≈57%) when [urea] is decreased from 8 to 0 M. However, for polyL we observed only ≈12% compaction for both N=16 and N=512 with increasing [TMAO] from 0 to 8 M. These results suggest that for hydrophilic polyQ chains, significant size compaction occurs in the presence of [TMAO] for larger chains. For hydrophobic polyL chains, a similar extent of compaction only occurs with decreasing [urea]. There is no significant change in $\langle R_{g}\rangle /\sqrt{N}$ in the presence of [TMAO] for longer polyL chains as they are already in the globular state (Fig. S11B). These observations are further corroborated by the structure factor plots (Figs. S12 and S13). We conclude that the extent of compaction/expansion in IDP size in the presence of cosolvents depends on the IDP length, residues (hydrophobic, hydrophilic, and charged) present in the IDP, nature of the cosolvent (stabilizing/destabilizing), and its concentration.

### Coil-to-globule crossover scaling with changes in added cosolvent concentration

At the *Θ*-temperature, for homopolymers the scaling relation (24, 72) $R_{g}^{2}=Nf(\tau \sqrt{N})$ holds, where $\tau =(T/T_{\Theta }-1)$, and *f* is a function of $\tau \sqrt{N}$. Here, we make the strong hypothesis that a similar scaling relation holds when cosolvents are used to modulate the solvent conditions, namely $R_{g}^{2}=Nf(c\sqrt{N})$, where $c=([C]/[C_{\Theta }]-1)$ and $\left[C_{\Theta }\right]$ is the cosolvent concentration at the *Θ*-point. Through this ansatz, we assume that adding the cosolvent changes the averaged effective monomer–monomer interaction strength, just like changing the temperature in a conventional polymer solution. This hypothesis is the central result of the article and should be relevant beyond the two specific IDPs we studied in this work. However, effects like co-nonsolvency (55) as mentioned above, are not described by such a model.

We plotted $R_{g}^{2}/N$ as a function of $c\sqrt{N}$ for polyQ and polyL for N≥128 and show that this scaling relation holds for the cosolvents (Fig. 4A and B). For short chains (N≤64) we observe deviations from this scaling in agreement with our results for pure water on the CG transition (Fig. S14). We identified $R_{g,\Theta }^{2}$ and $\left[C_{\Theta }\right]$ for polyQ and polyL from the plots where c=0. The $\left[C_{\Theta }\right]$ for polyQ and polyL are [TMAO]=2 M and [urea]=6.5 M, respectively. The values of $\langle R_{g,\Theta }^{2}\rangle /N$ for polyQ and polyL in the Θ−regime are 8 Å and 10 Å, respectively (Fig. 4A, B).

We probed the coil-globule transition for the IDP chains by computing the expansion factor, $\alpha_{R_{g}}(N)=\langle R_{g}^{2}\rangle /\langle R_{g,\Theta }^{2}\rangle$, where $\left\langle R_{g}^{2}\right\rangle$ and $\left\langle R_{g,\Theta }^{2}\right\rangle$ are the average radius of gyration for a chain of length *N* at a particular solvent condition, and *Θ*-solvent condition, respectively (Fig. 4C and D). We used the $\langle R_{g,\Theta }^{2}\rangle /N$ values obtained in the analysis described above to compute $\alpha_{R_{g}}$ for different *N*. If the polypeptide chain is in the *Θ*-regime, then $\alpha_{R_{g}}$ = 1, and deviation from *Θ*-regime, $\alpha_{R_{g}}>1$ (<1) denotes a good (poor) solvent condition. It is well established that the coil-globule transition in finite length homopolymers is not sharp, and there is a crossover region with $1/\sqrt{N}$ dependence (22, 23) (Fig. 2). Since polyQ is hydrophilic and exists as a SAW in water, we induced coil-globule transition by increasing [TMAO]. For long chains (N≥128), we observed that the $\alpha_{R_{g}}$ of polyQ monotonically increased for [TMAO]≤1 M indicating that the solvent conditions are good under these conditions (Fig. 4C). $\left[C_{\Theta }\right]$ for polyQ is estimated as [TMAO]=2 M, and $\alpha_{R_{g}}$ is ≈1 for N≥128 indicating that these chains are in the *Θ*-regime. For [TMAO]≥3 M, the polyQ is in poor solvent conditions as $\alpha_{R_{g}}<1$ for N≥128. Due to the intrinsic stiffness, we cannot infer the solvent quality for the short chains (N≤64) directly from this analysis. Since $\alpha_{R_{g}}(N)$ is still increasing with *N* for all [TMAO]≤4 M, a naive inference that these chains (N≤64) are in poor or good solvent conditions is misleading.

Similar behavior of $\alpha_{R_{g}}$ was observed for polyL with *N* for [urea]=0 to 8 M (Fig. 4D). The $\left[C_{\Theta }\right]$ for polyL is [urea]=6.5 M, and $\alpha_{R_{g}}$ is ≈1 for N≥128 indicating that these chains are in the *Θ*-regime (Fig. 4D). The longer chains (N≥128) are in good solvent condition when [urea]>6 M as $\alpha_{R_{g}}>1$. For shorter polyL chains (N≤64) due to the intrinsic stiffness, this analysis again is misleading because the chains appear to be in poor solvent for all [urea] as $\alpha_{R_{g}}<1$.

The scaling relation, $R_{g}^{2}=Nf(\tau \sqrt{N})$ proposed here, can be validated by measuring the second virial coefficient $B_{22}$ using osmometry experiments. The cosolvent concentration at which $B_{22}=0$ corresponds to the *Θ*-solvent concentration $\left[C_{\Theta }\right]$. Thus, comparing the $\left[C_{\Theta }\right]$ obtained from osmometry experiments with the $\left[C_{\Theta }\right]$ predicted by the scaling relation allows us to assess the applicability of the proposed scaling relation.

### Cosolvent concentration estimated for finite length IDP chains in the *Θ*-regime shows IDP property dependence

**Fig. 5.**
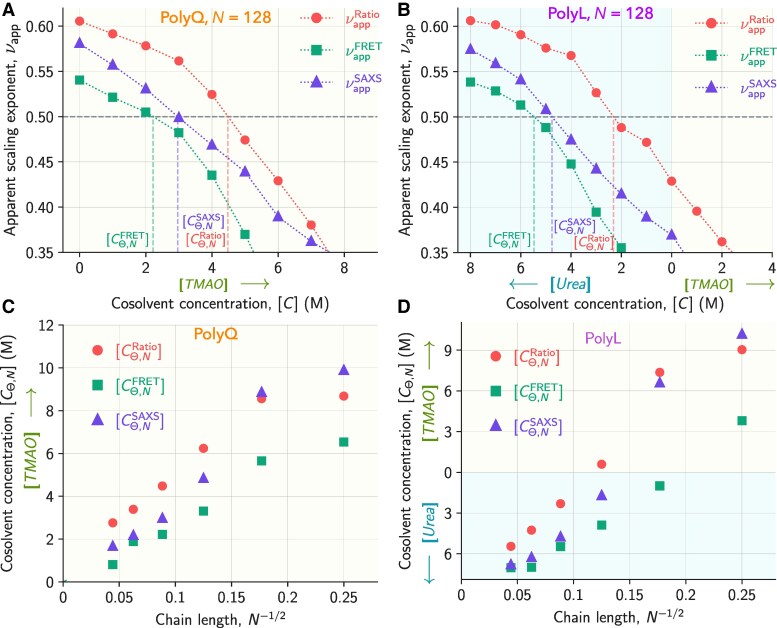
The apparent scaling exponent $\nu_{\mathrm{app}}$ is plotted as a function of cosolvent concentration [C] extracted using the three methods—critical ratio (red circles), pair distances mimicking FRET (green squares), and structure factor mimicking SAXS (violet triangles) for A) polyQ, N=128 and B) polyL, N=128. The gray horizontal dotted line denotes the CG transition (*Θ*-region), where $\nu_{\mathrm{app}}=1/2$. The three vertical dashed lines correspond to the coil-globule transition concentration $\left[C_{\Theta ,N}\right]$ from three different methods. The green and cyan shaded regions correspond to the cosolvents, TMAO and urea, respectively. Cosolvent concentration at the CG transition ($\left[C_{\Theta ,N}\right]$) for an IDP of length *N* as a function of $N^{-1/2}$ for C) polyQ and D) polyL calculated using three different methods mentioned earlier. PolyQ chains attain the *Θ*-regime at different [TMAO] depending on *N*. Whereas, polyL chains depending on *N* attain the *Θ*-regime at different [TMAO] and [urea].

The *Θ*-point is a tricritical point observed when N→∞. The finite length chains exhibit a crossover region known as the *Θ*-regime between the good solvent conditions where the IDP exhibits SAW, and the poor solvent conditions where globules are observed. Technically, using the relation $\log\;(\alpha_{R_{g}})=0$ we can identify the cosolvent concentration ($\left[C_{\Theta ,N}\right]$) for an IDP when it is in the *Θ*-regime and exhibits ideal chain-like behavior with the apparent scaling exponent, $\nu_{\mathrm{app}}=1/2$. However, the calculation of $\alpha_{R_{g}}$ requires $R_{g,\Theta }$ which is also a function of *N*. Therefore, to measure chain length dependent theta point, we define $\left[C_{\Theta ,N}\right]$ as the coil-globule transition cosolvent concentration where $\nu_{\mathrm{app}}=1/2$, and used three different methods for extracting $\nu_{\mathrm{app}}$ and $\left[C_{\Theta ,N}\right]$ (Fig. 5A and B, Supplementary materials S15 and S16): (a) plotting S(q) as a function of *q* and using the relation, $S(q)∼q^{-1/\nu }$ in the intermediate region, (b) plotting $\left\langle R_{\left|i-j\right|}\right\rangle$ as a function of |i−j| and using the relation $\langle R_{\left|i-j\right|}\rangle ∼|i-j{|}^{\nu }$, (c) using the critical ratio (39, 73), $\frac{\left\langle R_{\mathrm{ee}}^{2}\right\rangle }{\left\langle R_{g}^{2}\right\rangle }=\frac{2(\gamma +2\nu )(\gamma +2\nu +1)}{\gamma (\gamma +1)}$, where γ=1.1615. We labeled the cosolvent concentration as $\left[C_{\Theta ,N}\right]$, where we observed $\nu_{\mathrm{app}}=1$/2 using the above three methods (Fig. 5A and B and Supplementary materials S15 and S16). The value of $\nu_{\mathrm{app}}$ extracted using the critical ratio is labeled as $\nu_{\mathrm{app}}^{\mathrm{Ratio}}$. Similarly, the $\nu_{\mathrm{app}}$ value obtained from scattering intensity mimicking SAXS experiments and from multiple pair distances mimicking FRET experiments are labeled as $\nu_{\mathrm{app}}^{\mathrm{SAXS}}$ and $\nu_{\mathrm{app}}^{\mathrm{FRET}}$, respectively.

We observed that the $\nu_{\mathrm{app}}$ extracted for an IDP for all *N* using each method increased with an increase in [urea] due to the stabilization of extended states, whereas $\nu_{\mathrm{app}}$ decreased with an increase in [TMAO] due to the stabilization of compact states (Fig. 5A and B and Supplementary materials S15 and S16). However, $\nu_{\mathrm{app}}^{\mathrm{SAXS}}$, $\nu_{\mathrm{app}}^{\mathrm{FRET}}$, and $\nu_{\mathrm{app}}^{\mathrm{Ratio}}$ are different from each other for an IDP at a fixed [C] and follow the trend $\nu_{\mathrm{app}}^{\mathrm{Ratio}}>\nu_{\mathrm{app}}^{\mathrm{SAXS}}>\nu_{\mathrm{app}}^{\mathrm{FRET}}$ for all *N*. Since the $\nu_{\mathrm{app}}$ values obtained from the three methods differ, the coil-globule transition concentrations $\left[C_{\Theta ,N}\right]$ obtained using these methods are also different. $\left[C_{\Theta ,N}\right]$ obtained for polyQ using the three methods: $\left[C_{\Theta ,N}^{\mathrm{Ratio}}\right]$, $\left[C_{\Theta ,N}^{\mathrm{SAXS}}\right]$, and $\left[C_{\Theta ,N}^{\mathrm{FRET}}\right]$ ranges between [TMAO]=9–2.8M,10–1.5M, and 6.5–0.9 M, respectively with increasing *N* suggesting that the [TMAO] required for the coil-globule transition decreases with chain length (Fig. 5A and B and Supplementary materials S15 and S16). We also measured $\left[C_{\Theta ,N}\right]$ for polyL, which showed a similar trend to polyQ. Interestingly, the shorter polyL chains need TMAO (protective osmolyte), whereas the longer polyL chains need urea (denaturant) to reach the *Θ*-regime. This observation suggests that the nature of the cosolvent required for the IDP to be in the *Θ*-regime depends on *N*, and $\left[C_{\Theta ,N}\right]$ for a finite length IDP depends on the IDP property used to estimate it, chain length, and the residue type (hydrophobic, hydrophilic, and charged) in the IDP. In turn, this also means that for shorter chains the propensity to find LLPS can only be inferred from multiple chain simulations and as a function of IDP concentration.

### Cosolvent concentration for IDP in the *Θ*-regime scales with chain length

Previous studies (24, 37) on homopolymer established that the temperature ($T_{\Theta }^{N}$) for a finite length chain to be in the *Θ*-regime varies with chain length *N* and follows the scaling relation $T_{\Theta }^{N}-T_{\Theta }=mN^{-1/2}$, where $T_{\Theta }$ is the temperature at the *Θ*-point (tricritical point) for a long chain (N→∞) and *m* is a constant. In the previous sections, we showed that for both polyQ and polyL, the cosolvent concentration for coil-globule transition $\left[C_{\Theta ,N}\right]$ strongly depends on *N*. We plotted $\left[C_{\Theta ,N}\right]$ for polyL and polyQ as a function of *N* to probe the scaling relation between these quantities (Fig. 5C and D).

We observed that $\left[C_{\Theta ,N}\right]$ varies linearly with $N^{-1/2}$ for polyQ, i.e. the required [TMAO] for coil-globule transition decreases with increasing *N* (Fig. 5C). However, the slope (*m*) of $\left[C_{\Theta ,N}\right]$ vs. *N* plot is property-dependent. *m* from various properties follow the same pattern: $m^{\mathrm{Ratio}}>m^{\mathrm{SAXS}}>m^{\mathrm{FRET}}$. Additionally, $\left[C_{\Theta ,N}\right]$ from all three properties appears to converge at a single concentration only at N→∞ (Fig. 5C). This observation is in agreement with the previous studies on homopolymers (24, 37). We observed that for a finite-sized IDP, $\left[C_{\Theta ,N}^{\mathrm{Ratio}}]>[C_{\Theta ,N}^{\mathrm{SAXS}}]>[C_{\Theta ,N}^{\mathrm{FRET}}\right]$ is valid for most cases. The different $\left[C_{\Theta ,N}\right]$ values obtained from different properties measured using different experiments might lead to a disagreement regarding the IDPs behavior. For a fixed cosolvent concentration, since $\nu_{\mathrm{app}}^{\mathrm{SAXS}}>\nu_{\mathrm{app}}^{\mathrm{FRET}}$, we could arrive at misleading conclusions regarding the solvent quality from the SAXS and FRET experiments if $\nu_{\mathrm{app}}^{\mathrm{SAXS}}>0.5$ and $\nu_{\mathrm{app}}^{\mathrm{FRET}}<0.5$.

The slope *m* estimated from various properties is different because the finite-size corrections to extract the universal scaling exponent *ν* valid for large *N* are property dependent (39, 40). For polymers, *ν* is generally extracted by measuring $R_{g}$, $R_{\mathrm{ee}}$, and $R_{h}$ from SAXS, single molecular fluorescence resonance energy transfer, and FCS experiments, respectively. In addition to *ν*, even the ratios $\left\langle R_{\mathrm{ee}}^{2}\rangle /\langle R_{g}^{2}\right\rangle$ and $\left\langle R_{g}^{2}\rangle^{1/2}/\langle R_{h}\right\rangle$ are universal depending on the solvent condition in the limit N→∞. The slope *m* for a property depends on how strong the corrections to the scaling are and they are given by (39, 74)


(2) $$\left\langle R_{g}^{2}\rangle =D_{g}N^{2\nu }(1+a_{g}N^{-\Delta_{1}}+\cdots \right)$$

(3) $$\left\langle R_{\mathrm{ee}}^{2}\rangle =D_{e}N^{2\nu }(1+a_{e}N^{-\Delta_{1}}+\cdots \right)$$

(4) $$\left\langle R_{h}^{-1}\rangle =D_{h}N^{-\nu }(1+a_{h}N^{-\Delta_{1}}+b_{h}N^{-(1-\nu )},+\cdots \right)$$

where the primary correction to the scaling exponent is $\Delta_{1}$ (≈0.51), and $D_{g}$, $D_{e}$, $D_{h}$, $a_{g}$, $a_{e}$, and $a_{h}$ are nonuniversal amplitudes. The above equations further show that scaling corrections to $R_{h}$ are stronger due to the $N^{-(1-\nu )}$ term.

Previous studies (24, 36, 37) reported the relation $T_{\Theta }^{N}-T_{\Theta }=mN^{-1/2}$ in temperature-length (*T*-*N*) space and provided evidence that the slope *m* can be positive or negative, i.e. above or below the *Θ* temperature, respectively. Generally, increasing *T* is correlated with decreasing the effective solvent-mediated monomer–monomer interaction strength $\epsilon_{\mathrm{mm}}∼1/T$. If water is solvent, this relation could be complex due to the *T*-dependent hydrophobic effect arising due to the unique properties of water (75, 76). These effects are known to also lead to the LCST (lower critical solution temperature) behavior for many (bio-)polymers, which is not the subject of the current study (23). On the other hand, increasing the cosolvent concentration can also modulate the effective $\epsilon_{\mathrm{mm}}$ depending on the type of cosolvent i.e. $\epsilon_{\mathrm{mm}}∼1/[\mathrm{urea}]$ or $\epsilon_{\mathrm{mm}}∼[\mathrm{TMAO}]$. As a result, we also observed similar behavior for polyL chains, where $\left[C_{\Theta ,N}\right]$ varies as $N^{-1/2}$ (Fig. 5D). The $\left[C_{\Theta ,N}\right]$ estimated using the three methods mentioned earlier differ for finite-sized chains and converge to a single value for long chains, similar to homopolymers and polyQ. The dependency of $\left[C_{\Theta ,N}\right]$ on *N* quantified by the slope of $\left[C_{\Theta ,N}\right]$ vs. $N^{-1/2}$ plot shows a similar trend as polyQ: $\left[C_{\Theta ,N}^{\mathrm{Ratio}}]>[C_{\Theta ,N}^{\mathrm{SAXS}}]>[C_{\Theta ,N}^{\mathrm{FRET}}\right]$ for finite size polyL chains (Fig. 5D). For longer polyL chains, the denaturant urea is required to reach the *Θ*-regime (Fig. 5D). For shorter polyL chains, the naive inference is the protective osmolyte, TMAO is required to attain the *Θ*-regime (Fig. 5D), which is misleading as shorter polyL chains exhibit SAW behavior independent of the solvent quality due to intrinsic stiffness. Hence, multichain simulations should be performed for short chains to infer the aggregation behavior.

#### LLPS propensity and droplet size increase with chain length

**Fig. 6.**
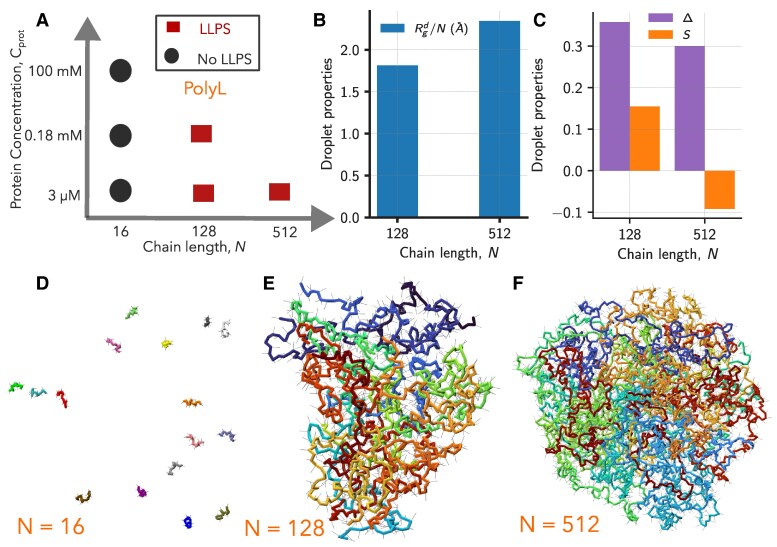
A) Droplet formation by polyL for different protein concentrations ($C_{\mathrm{prot}}$) and chain lengths (*N*). Red squares (black circles) denote the presence (absence) of the droplet. B) The rescaled radius of gyration ($R_{g}^{d}/N$) and C) asphericity (*Δ*) and shape (*S*) of the polyL droplets as a function *N*. Representative snapshots from the multichain simulations of polyL chains, D) N=16, E) N=128, and F) N=512.

In the previous sections, we showed that for short chains, due to intrinsic stiffness, which is independent of the solvent quality, we cannot infer the multichain behavior from single-chain properties. A recent study (33) has also demonstrated that the arrangement of amino acid residues in IDP sequences, specifically local vs. nonlocal sequence patterns, can also influence the correlation between single-chain and multichain behaviors of IDPs. For longer chains, the thermal blob size depends on the solvent quality, and we can infer the multichain behavior from the single chain properties as a function of solvent quality. Therefore, to verify the chain length dependence of polyL on LLPS, we performed multichain simulations for N=16, 128, and 512. For all the systems, we fixed the number of polyL chains to 16 in the simulation box and varied the box length from 70 to 2050 Å to mimic polyL concentrations $C_{\mathrm{prot}}=3\;\mu M$, 0.18 mM, and 100 mM (Fig. 6A). For N=16, we did not observe any droplet formation, and polyL chains are in the dilute phase for all the $C_{\mathrm{prot}}$ (Fig. 6A and D). However, for both N=128 and 512, we observed droplet formation for all $C_{\mathrm{prot}}$ (Fig. 6A, E, and F). Further, when *N* increased from 128 to 512, the radius of gyration of the droplet ($R_{g}^{d}$) (see Materials and methods) rescaled with *N* increased from 1.8 to 2.3 Å suggesting that the droplet size increases with *N* (Fig. 6B). The results suggest that the critical IDP concentration required for LLPS decreases, and the stability of droplets increases with *N*, in agreement with the previous experiments (34, 77–79), simulations (80) and the general theoretical phase diagram (Fig. 2A). The dependency of polyL and polyQ on *N* as $N^{-1/2}$ to approach the *Θ*-regime indicates that the critical concentration for phase separation will also vary as $N^{-1/2}$, in agreement with the homopolymer theory (37).

The results from this study are relevant to understand the properties of other biomolecules such as (deoxy) ribonucleic acids (DNAs/RNAs). Homopolymeric RNA (DNA) sequences such as ${\mathrm{rA}}_{N}$ (${\mathrm{dA}}_{N}$) and ${\mathrm{rU}}_{N}$ ($dT_{N}$) are used to facilitate the phase separation phenomenon in vitro. Experiments (81) probing the dependence of shorter sequence length (10≤N≤30) on the LLPS showed that the critical concentration of divalent cations required to induce the phase separation decreases with an increase in *N*, which further confirms *N* dependent LLPS propensity of the nucleic acids.

#### Chain rigidity influences the morphology of the protein condensates

The structural morphology of the aggregates formed by polypeptide chains depends on their stiffness (82). Stiff polypeptide chains adopt an extended configuration at the single molecule level and can form aggregates in which the monomers are extended, resulting in high asphericity of the aggregate. In contrast, polypeptide chains with lower stiffness tend to form globules in poor solvent conditions, and the aggregates adopt globule shapes with low asphericity.

Since the intrinsic stiffness of a chain is independent of the chain length, we expect chains with large *N* to form spherical droplets (low asphericity) compared to chains with small *N*. The multichain simulations show that with *N* increasing from 128 to 512, the asphericity *Δ* (see Methods) decreases from ≈0.35 to 0.3, suggesting the formation of more spherical droplets for larger *N* (Fig. 6C–E). Moreover, the shape of the droplet *S* (see Methods) also varies from a prolate (S=0.15) to oblate (S=−0.09) with *N* increasing from 128 to 512 (Fig. 6C), as stiffer short chains aggregate to form droplets with shape needle-like prolate ellipsoids.

## Conclusion

IDPs are finite-length heteropolymers and are studied using various experimental techniques to probe their properties spanning multiple time scales (9, 10, 41–44, 49, 50). The experimental data are routinely analyzed using homopolymer theory valid for infinite-length chains (22, 23). Furthermore, it is essential to understand the intricacies of using polymer theory to analyze the data obtained from different experimental techniques to infer an IDPs’ *Θ*-regime.

We studied the dependence of *Θ*-regime induced by the cosolvents on the IDP chain length using computer simulations and coarse-grained IDP models (21). The IDPs we used, polyQ and polyL, are homopolymers to arrive at the rigorously established results. The important results of this study are that IDP chains of length equivalent to their intrinsic stiffness exhibit self-avoiding walk like behavior irrespective of the solvent quality, and their aggregation propensity cannot be inferred from the single-chain properties. Multichain simulations in different solvent conditions should be performed to understand the aggregation of these IDPs. The cosolvent concentration required for the IDP of length *N* to be in the *Θ*-regime varied as $N^{-1/2}$ and reached the limiting value $C_{\Theta }$ (*Θ*-point) only when N→∞. In addition to *N*, the type of amino acids present in the IDP modulates the cosolvent concentration and the type of cosolvent required for the coil-globule transition (9, 50). A consequence of the dependence of the *Θ*-regime on the chain length is that the propensity of the IDP to exhibit LLPS increases with the chain length.

We further show that the *Θ*-regime inferred using SAXS and FRET experimental techniques, which measure $R_{g}$ and $R_{\mathrm{ee}}$, differed for finite-length chains and converged only for long chains. As a result, there could be disagreements (41–44, 49, 50, 83, 84) in the conclusions of these experimental techniques regarding the finite-length IDP states. The main result of this article is that we proposed a scaling relation for the IDPs valid in the *Θ*-regime induced by the cosolvents. Different experimental techniques, which measure $R_{g}$, $R_{\mathrm{ee}}$, and $R_{h}$ of an IDP, can use the proposed scaling relation to unambiguously extract the cosolvent concentration for the IDP to be in the *Θ*-regime and the IDP size in the *Θ*-regime.

Finally, we point out the limitations of the coarse-grained model used in the simulations. We have used the Self-Organized Polymer (SOP)-IDP (21) model, which reasonably reproduced the experimental $R_{g}$ values and SAXS profile for 24 different IDPs. However, due to the reduced degrees of freedom in the coarse-grained models, SOP-IDP model may fail to capture the subtle balances in the energetic interactions between the various residues and solvents under all conditions. As a result, the estimation of the solvent quality for a polymer, which depends on the rate at which the size of the polymer increases with the chain length, might not be precisely captured. Also, since the amino acids are approximated to only two beads in the model, the intrinsic stiffness, which depends on the atomistic details, can only be approximately estimated. However, we point out that these limitations do not affect the conclusions of this work. In this work, we aim to understand the limitations of various experimental methods used in estimating the *Θ*-regime of finite-sized IDPs induced by the cosolvents. We proposed a scaling relation to precisely calculate the cosolvent concentration for the finite-size IDP in the *Θ*-regime, and these results are independent of the IDP models.

This work primarily focuses on the deviations in polymer scaling behavior due to the finite size of homopolypeptides such as polyQ and polyL. However, IDPs are generally heteropolymers, and their conformational ensemble could be highly heterogeneous, which could also lead to deviations from the scaling behavior observed in long homopolymers (50, 84, 85).

## Materials and methods

We used a two-bead coarse-grained self-organized polymer model for IDPs (SOP-IDP) (21) model to study the properties of IDPs. The first bead represents the backbone atoms, and the second bead represents the side-chain atoms (Fig. 1A). The energy function of an IDP with bead coordinates {r}, in the absence of a cosolvent ([C]=0) is comprised of three terms: bonded interactions ($E_{B}$), local nonbonded interactions ($E_{\mathrm{NB}}^{L}$), and nonlocal nonbonded interactions ($E_{\mathrm{NB}}^{\mathrm{NL}}$) and is given by $E(\left\{r\right\},0)=E_{B}+E_{\mathrm{NB}}^{L}+E_{\mathrm{NB}}^{\mathrm{NL}}$. In the multichain simulations, we introduced the interchain interaction, $E_{\mathrm{inter}}$, which accounts for the interaction between beads of two different chains. The parameters in the SOP-IDP model are optimized using the SAXS profile data of 24 different IDPs. The SOP-IDP model and energy function for multichain simulations are explained in detail in the Supplementary material.

We introduced the cosolvent effect on the IDP using molecular transfer model (MTM) where the modified energy function of a protein conformation with bead coordinates ({r}) in a cosolvent solution with concentration ([C]) is given by $E(\left\{r\right\},[C])=E(\left\{r\right\},0)+\Delta G_{tr}(\left\{r\right\},[C])$, where $\Delta G_{tr}(\left\{r\right\},[C])$ is the free energy difference for transferring a protein conformation given by bead coordinates {r} from water to a cosolvent solution of concentration [C]. Details of the MTM model are described in the Supplementary material.

To infer the intrinsic stiffness, we performed Langevin dynamics simulations for NRRW with chain length, N=256 at T=300 K using the modified energy function of SOP-IDP model, $E_{\mathrm{NRRW}}(\left\{r\right\})=E_{B}+E_{\mathrm{NB}}^{L}+E_{\mathrm{NB}}^{\mathrm{mNL}}$, where $E_{B}$ and $E_{\mathrm{NB}}^{L}$ energy function and parameters are same as SOP-IDP model for polyQ and polyL for N=256. $E_{\mathrm{NB}}^{\mathrm{mNL}}$ is the interaction term that only exists if the pair of beads is within three to six residues apart.

We performed low friction Langevin dynamics simulations in the presence of TMAO (0–8 M) and urea (0–8 M) for polyQ and polyL with N=16–512. We characterized the IDP conformations using the radius of gyration, $R_{g}^{2}=(1/2N_{\mathrm{res}}^{2})\sum_{i,j}{\vec{r}}_{ij}^{2}$, hydrodynamic radius, $R_{h}^{-1}=(1/N_{\mathrm{res}}^{2})\sum_{i\neq j}(1/|{\vec{r}}_{ij}|)$, and end-to-end distance, ${\vec{R}}_{\mathrm{ee}}=\sum_{i}{\vec{r}}_{i,i+1}$, where $N_{\mathrm{res}}$ is the number of residues in the IDP, $r_{ij}$ is the distance between the backbone beads of residues *i* and *j*, and the summation is over all the backbone beads. We also calculated $\left\langle R_{g}\right\rangle$  $\left(={\left(\frac{1}{2n^{2}}\sum_{i=0}^{n-1}\sum_{\;j=i+1}^{n}{\vec{r}}_{ij}^{2}\right)}^{1/2}\right)$ and $\left\langle R_{\mathrm{ee}}\right\rangle$  $\left(=\left|\sum_{i=0}^{n-1}{\vec{r}}_{i,i+1}\right|\right)$ as a function of segment length *n* for the IDPs. The normalized structure factor (23), S(q), is calculated using the equation, $S(q)=\frac{1}{N_{\mathrm{res}}}\sum_{i=1}^{N_{\mathrm{res}}}\sum_{\;j=1}^{N_{\mathrm{res}}}\frac{\sin\;(qr_{ij})}{qr_{ij}}$, where *q* is the wave vector. The simulation timescales are much longer than the decay time of the $R_{\mathrm{ee}}$ autocorrelation function to ensure good conformational sampling to compute the equilibrium properties (Figs. S17 and S18).

The radius of gyration of the droplet is given by $R_{g}^{d}={\left(\frac{1}{2(N_{\mathrm{res}}\times N_{\mathrm{ch}}{)}^{2}}\sum_{i,j}^{N_{\mathrm{res}}\times N_{\mathrm{ch}}}{{\vec{r}}_{ij}}^{2}\right)}^{1/2}$, where $N_{\mathrm{ch}}$ is the number of chains in the droplet. Asphericity (*Δ*) and shape (*S*) of the droplet are computed using the definition, $\Delta =\frac{3}{2}\frac{(\lambda_{i}-\langle \lambda \rangle {)}^{2}}{(3\langle \lambda \rangle {)}^{2}}$ and $S=\Pi_{i=1}^{3}\frac{\lambda_{i}-\langle \lambda \rangle }{\langle \lambda \rangle^{3}}$, where $\lambda_{i}$ are the eigenvalues of the gyration tensor of the droplet and $\langle \lambda \rangle =\left(\sum_{i}\lambda_{i}\right)/3$.

## Supplementary Material

pgaf039_Supplementary_Data

## Data Availability

All the generated data are in the article and Supplementary material file. The codes are available at https://doi.org/10.5281/zenodo.14766437.
