# Correction to: Intrinsic stiffness and *Θ*-solvent regime in intrinsically disordered proteins: Implications for liquid–liquid phase separation

**DOI:** 10.1093/pnasnexus/pgaf383

**Published:** 2026-03-18

This is a correction to: Lipika Baidya, Kurt Kremer, Govardhan Reddy, Intrinsic stiffness and *Θ*-solvent regime in intrinsically disordered proteins: Implications for liquid–liquid phase separation, *PNAS Nexus*, Volume 4, Issue 2, February 2025, https://doi.org/10.1093/pnasnexus/pgaf039

In this article, the *x*-axis of structure factor S(q) plots in the main manuscript (Fig. 3B,D), and in the supporting information (Fig. S2A, S9A, S12 and S13) were mistakenly labeled as inverse of nanometer (nm^−1^) instead of inverse angstroms (Å^−1^). Because of this mistake, the *x*- and *y*-axis labels of the SI figures, Figs. S2B and S9B, should read 0.1 × $qR_{g}$ and 0.01 × $\left(qR_{g}{)}^{2}S(q\right)$, respectively, instead of $qR_{g}$ and $\left(qR_{g}{)}^{2}S(q\right)$.

Due to the mistake mentioned above, in the subsection *“Intrinsic stiffness in finite length PolyQ and PolyL chains contributes to contrasting behavior compared to the long polymer chains”* in the main manuscript, we wrongly concluded that on length scales q≈0.4 to 1.0 nm^−1^, the S(q) for all *N* collapses onto a single curve due to intrinsic stiffness, indicating that the solvent quality is the same for all chain lengths on this short length scale, and inferred that the intrinsic stiffness extends to about 10 to 25 residues for polyglutamine and polyleucine chains. The correct inference for this subsection should be that on length scales q≈0.4 to 1.0 Å^−1^, the S(q) for all *N* collapses onto a single curve due to intrinsic stiffness, and intrinsic stiffness only extends to about 1 to 3 residues for polyglutamine and polyleucine chains.

These corrections are limited to unit representation on the *x*-axis of S(q) plots. The underlying data, analysis, and conclusions for the other parts of the study remain unaffected by this correction.

We sincerely apologize for any confusion this may have caused and thank readers for their understanding.

