# Supplementary material for: Intrinsic stiffness and Θ-solvent regime in intrinsically disordered proteins: Implications for liquid–liquid phase separation
Source: PNAS Nexus. 2025 Feb 5;4(2):pgaf039. doi: 10.1093/pnasnexus/pgaf039 (PMC11840863; doi:10.1093/pnasnexus/pgaf039)
Supplement: pgaf039_Supplementary_Data [file pgaf039_supplementary_data.pdf]

PNAS nexus

Supplementary Information for

Intrinsic Stiffness and Θ-Solvent Regime in the Intrinsically Disordered Proteins: Implications for the Liquid-Liquid Phase Separation

Lipika Baidya, Kurt Kremer and Govardhan Reddy

Govardhan Reddy
E-mail:greddy@iisc.ac.in

This PDF file includes:

Supplementary text
Figs. S1 to S19 (not allowed for Brief Reports)
Tables S1 to S2 (not allowed for Brief Reports)
SI References

Supporting Information Text

Materials and Methods

Self Organized Polymer Model for IDPs (SOP-IDPs) . We performed the simulations using a self-organized polymer model for intrinsically disordered proteins (SOP-IDP) described elsewhere(1). In the SOP-IDP model, each residue is represented as two beads - one bead for the backbone atoms and the other bead for side chain atoms. The backbone and side chain beads are located at C_α position and the center of mass of side chain atoms, respectively. We generated an elongated chain with the polypeptide backbone beads pointed along the Z-direction to get the initial coarse-grained coordinates ($\{\mathbf{r}\}$) for the polypeptides.

The energy function ($E_{CG}(\{\mathbf{r}\},0)$) of the SOP-IDP model in the absence of cosolvent is the sum of bonded (E_B) and non-bonded (E_{NB}) interactions. The non-bonded energy consists of local (E^L_{NB}) and non-local (E^{NL}_{NB}) interactions. The Hamiltonian of the SOP-IDP model is

$$E_{CG}(\{\mathbf{r}\},0) = E_B + E^L_{NB} + E^{NL}_{NB} \quad \text{[S1]}$$

The interaction between two bonded beads (E_B) is modeled using the finite extensible non-linear elastic (FENE) potential,

$$E_B = -\sum_{i=1}^{N_B} \frac{k}{2} R_0^2 \log\left(1 - \frac{(r_i - r_i^0)^2}{R_0^2}\right), \quad \text{[S2]}$$

where N_B is the total number of bonds in the SOP-IDP model. r_i is the instantaneous bond distance between the ith pair of bonded beads and r_i^0 is the corresponding equilibrium bond distance, and R_0 is the maximum bond extension/compression. The values of r_i^0 are set to the sum of van der Waals radii. The values of k and R_0 are given in Table S1.

The two beads, which are not connected by a covalent bond and separated by less than three residues along the polypeptide chain, interact with each other through a nonbonded local potential (E^L_{NB}). E^L_{NB} is modeled with a purely repulsive potential accounting for excluded volume interactions to prevent unphysical overlap between the two non-bonded beads and is given by

$$E^L_{NB} = \sum_{i=1}^{N_l} \epsilon_l \left(\frac{\sigma_i}{r_i}\right)^6, \quad \text{[S3]}$$

where σ_i is the sum of the van der Waals (vdW) radii of ith pair of non-bonded beads and ϵ_l is the strength of repulsive interaction. The value of ϵ_l is given in Table S1, and values of vdW radii for each amino acid residue are listed in Table S2.

The beads, separated by more than two residues, interact through non-bonded non-local interaction, E^{NL}_{NB}, and are modeled using Lenard-Jones potential.

$$\begin{aligned} E^{NL}_{NB} &= \sum_{i=1}^{N_{bb}} \epsilon_{bb}\left[\left(\frac{\sigma^{bb}}{r_i}\right)^{12} - 2\left(\frac{\sigma^{bb}}{r_i}\right)^6\right] \\ &+ \sum_{i=1}^{N_{bs}} \epsilon_{bs}\left[\left(\frac{\sigma_i^{bs}}{r_i}\right)^{12} - 2\left(\frac{\sigma_i^{bs}}{r_i}\right)^6\right] \\ &+ \sum_{i=1}^{N_{ss}} \epsilon_{ss} \mid 0.7 - \epsilon_i \mid \left[\left(\frac{\sigma_i^{ss}}{r_i}\right)^{12} - 2\left(\frac{\sigma_i^{ss}}{r_i}\right)^6\right]. \end{aligned} \quad \text{[S4]}$$

The first, second, and third terms of Eq. S4 correspond to the backbone-backbone, backbone-side chain, and side chain-side chain interaction energies, respectively. N_{bb}, N_{bs}, and N_{ss} denote the number of interaction pairs present between backbone-backbone, backbone-side chain, and side chain-side chain beads, respectively. r_i is the distance between i^{th} pair of beads. σ^{bb} is the diameter of the backbone bead, which is taken as 3.8 Å. σ_i^{bs} and σ_i^{ss} are the sum of bead radii for the ith pair of backbone-side chain and side chain-side chain beads, respectively. σ_i^{bs} and σ_i^{ss} are computed using the bead radii listed in Table S2. ϵ_{bb}, ϵ_{bs}, and ϵ_{ss} are the strength of backbone - backbone, backbone - side chain, and side chain - side chain interactions, respectively. We used the Betancourt - Thirumalai statistical potential(2) for ϵ_i to model the side chain - side chain interaction for amino acid pair i (Table S2).

For the multi-chain simulations, we added an interchain interaction term, E_{inter} and the effective energy function is

$E_{multi} = E_{CG}(\{\mathbf{r}\}, 0) + E_{inter}$. Interchain interaction is defined as

$$
\begin{aligned}
E_{inter} &= \sum_{i=1}^{N_{bb}^{inter}} \epsilon_{bb} \left[\left(\frac{\sigma^{bb}}{r_i} \right)^{12} - 2 \left(\frac{\sigma^{bb}}{r_i} \right)^{6} \right] \\
&+ \sum_{i=1}^{N_{bs}^{inter}} \epsilon_{bs} \left[\left(\frac{\sigma_i^{bs}}{r_i} \right)^{12} - 2 \left(\frac{\sigma_i^{bs}}{r_i} \right)^{6} \right] \\
&+ \sum_{i=1}^{N_{ss}^{inter}} \epsilon_{ss} \mid 0.7 - \epsilon_i \mid \left[\left(\frac{\sigma_i^{ss}}{r_i} \right)^{12} - 2 \left(\frac{\sigma_i^{ss}}{r_i} \right)^{6} \right]. \qquad \text{[S5]}
\end{aligned}
$$

N_{bb}^{inter}, N_{bs}^{inter} and N_{ss}^{inter} are the number of backbone-backbone, backbone-side chain and side chain-side chain pairs that are present in two different chains.

The ϵ_{bb}, ϵ_{bs}, and ϵ_{ss} parameters in the SOP-IDP model are optimized using the SAXS data for 24 IDPs and the values are 4.6, 1.7, and 1.7 times weaker, respectively, compared to the parameters used in the SOP-SC model to study folding of globular proteins(3, 4). In the SOP-SC model, to mimic the funnel-shaped free energy landscape of globular proteins, native contacts between the backbone and side chain beads are present based on their folded crystal structure. Meanwhile, in the SOP-IDP model, all possible non-local contacts between the beads (separated by three or more residues) are allowed to reflect the heterogeneous and flat free energy landscape of IDPs. Consequently, the pairwise interaction strengths in the SOP-IDP model (0.12–0.18 k_BT) are weaker than the native-like interactions in the SOP-SC model.

Molecular Transfer Model (MTM). To introduce the effect of cosolvent, we used the molecular transfer model (MTM), which was used extensively to study the folding thermodynamics of globular proteins(5). In MTM, the updated energy function of an IDP conformation with coordinates $(\{\mathbf{r}\})$ and cosolvent concentration $[C]$ is given by

$$E_{CG}(\{\mathbf{r}\}, [C]) = E_{CG}(\{\mathbf{r}\}, 0) + \Delta G_{tr}(\{\mathbf{r}\}, [C]), \qquad \text{[S6]}$$

where $\Delta G_{tr}(\{\mathbf{r}\}, [C])$ is the transfer free energy on transferring of an IDP conformation from water to a cosolvent solution with concentration $[C]$ and is given by

$$\Delta G_{tr}(\{\mathbf{r}\}, [C]) = \sum_{i=1}^{N_{res}} \delta g_{tr}^{bb}([C]) \frac{\alpha_i^{bb}(\{\mathbf{r}\})}{\alpha_{Gly-i-Gly}^{bb}} + \sum_{i=1}^{N_{res}} \delta g_{tr,i}^{sc}([C]) \frac{\alpha_i^{sc}(\{\mathbf{r}\})}{\alpha_{Gly-i-Gly}^{sc}}, \qquad \text{[S7]}$$

where N_{res} is the total number of residues in the IDP, $\delta g_{tr}^{bb}([C])$ and $\delta g_{tr,i}^{sc}([C])$ are the transfer free energies of backbone bead and side chain bead of ith residue from water to a cosolvent solution $[C]$, respectively. $\alpha_i^{bb}(\{\mathbf{r}\})$ and $\alpha_i^{sc}(\{\mathbf{r}\})$ are the solvent accessible surface area (SASA) of the backbone and side chain bead of residue i in the protein chain. $\alpha_{Gly-i-Gly}^{bb}$ and $\alpha_{Gly-i-Gly}^{sc}$ are the SASA of the backbone and side chain beads of the same amino acid residue i in the tripeptide $Gly-i-Gly$. The values of transfer-free energies and SASA of tripeptides are taken from Table S1 of Ref. (6) and Table S4 of Ref. (3). The van der Waals radius of water is taken as 1.4 Å. We used the approximate analytical method discussed in ref. (7) to calculate the SASA of the protein chain. Since MTM is an implicit solvent model, it cannot be used to study the co-nonsolvency or co-solvency effect, which might appear in mixed good or poor solvents. However, we have not studied these effects in this paper.

Simulation Details and Data Analysis. We carried out low friction Langevin dynamics(8) simulation at temperature, $T = 300$ K in the presence of cosolvent to compute the average thermodynamic properties of IDPs. The equation motion in Langevin dynamics is given by,

$$m\ddot{\vec{r}} = -\zeta\dot{\vec{r}} + \vec{F}_C + \vec{\Gamma}, \qquad \text{[S8]}$$

where m is the mass of protein beads, ζ is the friction coefficient of the solvent medium, $\vec{F}_C$ is the deterministic force given by $-\frac{\partial E_{CG}(\{\mathbf{r}\},[C])}{\partial \vec{r}_i}$, and $\vec{\Gamma}$ is the random force with Gaussian noise characterized by $\left\langle \vec{\Gamma}(t) \cdot \vec{\Gamma}(t+nh) \right\rangle = \frac{2\zeta k_B T}{h}\delta_{0,n}$ where $n = 0, 1, \cdots$, $\delta_{0,n}$ is the Kronecker delta function and k_{B} is the Boltzmann constant. We integrated Eq. S8 using the velocity Verlet algorithm. We used $\zeta = 0.05\ m/\tau_{\mathrm{LD}}$ and an integration time step, $h = 0.005\ \tau_{\mathrm{LD}}$, where, $\tau_{\mathrm{LD}} \left(= \sqrt{\frac{ma^2}{\epsilon_h}} \right)$ is the unit of time used to advance the Langevin dynamics simulations. The average mass of each bead (m), the characteristic unit of length (a), and energy (ϵ_h) are taken as 1.8 $\times 10^{-22}$ g, 1 Å, and 1 kcal/mol. The value of τ_{LD} in real time units is $\approx$ 1.3 ps.

We calculated the normalized structure factor(9), $S(q)$, of the IDP using the backbone beads, and it is given by

$$S(q) = \frac{1}{N_{res}} \sum_{i=1}^{N_{res}} \sum_{j=1}^{N_{res}} \frac{\sin(qr_{ij})}{qr_{ij}} \qquad \text{[S9]}$$

where q is the wave vector, N_{res} is the number of residues in the IDP and r_{ij} is the distance between the backbone beads of residues i and j. The radius of gyration, R_g, of the IDPs is given by

$$R_g = \left(\frac{1}{2N_{res}^2} \sum_{i,j} \vec{r}_{ij}^2 \right)^{1/2} \qquad \text{[S10]}$$

where $\vec{r}_{ij}$ is the vector joining beads i and j and N_{res} is the total number of residues in the IDP. The radius of gyration of the droplet, R_g^d, is given by

$$R_g^d = \left(\frac{1}{2(N_{res} * N_{ch})^2} \sum_{i,j}^{N_{res} * N_{ch}} \vec{r}_{ij}^2 \right)^{1/2} \qquad \text{[S11]}$$

where N_{ch} is the number of chains in the droplet.

Scattering by a Hard Sphere in Solution. For a dilute solution of hard spheres of radius R, where the spheres do not interact with each another and have a sharp density difference at the interface of the sphere and the solvent medium, the intensity of the scattered wave due to the sphere at the wave vector q is given by(10)

$$I(q) \sim \left[3 \left(\frac{\sin(qR) - qR\cos(qR)}{(qR)^3} \right) \right]^2 \qquad \text{[S12]}$$

We obtain the Porod scaling in the limit $qR \gg 1$, where Eq. S12 reduces to

$$I(q) \sim \frac{\cos^2(qR)}{(qR)^4} \qquad \text{[S13]}$$

due to which we observe oscillations in the scattered intensity, and $I(q) \sim q^{-4}$. In the limit $qR \ll 1$, Eq. S12 reduces to the form

$$I(q) \sim 1 - \frac{(qR)^2}{10} \qquad \text{[S14]}$$

Table S1. Parameters used in SOP-IDP Model

Parameter	Value
k	20.0 kcal/(mol.Å^2)
R_0	2.0 Å
ϵ_l	1.0 kcal/mol
ϵ_{bb}	0.12 kcal/mol
ϵ_{bs}	0.24 kcal/mol
ϵ_{ss}	0.18 kcal/mol

Table S2. Parameters of bead radius

Bead	vdW radius (Å)	ϵ_i (kcal/mol)
C_α	1.90	-
Gln (Q)	3.01	0.18
Leu (L)	3.09	-0.81

References

1. U Baul, D Chakraborty, M Mugnai, J Straub, D Thirumalai, Sequence effects on size, shape, and structural heterogeneity in intrinsically disordered proteins. *J. Phys. Chem. B* **123**, 3462–3474 (2019).
2. M Betancourt, D Thirumalai, Dissecting ubiquitin folding using the self-organized polymer model. *Protein Sci.* **8**, 361–369 (1999).
3. Z Liu, G Reddy, E O'Brien, D Thirumalai, Collapse kinetics and chevron plots from simulations of denaturant-dependent folding of globular proteins. *Proc. Natl. Acad. Sci. USA* **108**, 7787–7792 (2011).
4. G Reddy, Z Liu, D Thirumalai, Denaturant-dependent folding of GFP. *Proc. Natl. Acad. Sci. U.S.A.* **109**, 17832–17838 (2012).
5. E O'Brien, G Ziv, G Haran, B Brooks, D Thirumalai, Effects of denaturants and osmolytes on proteins are accurately predicted by the molecular transfer model. *Proc. Natl. Acad. Sci. USA* **105**, 13403–13408 (2008).
6. M Auton, D Bolen, Predicting the energetics of osmolyte-induced protein folding/unfolding. *Proc. Natl. Acad. Sci. U. S. A.* **102**, 15065–15068 (2005).
7. S Wodak, J Janin, Analytical approximation to the accessible surface area of proteins. *Proc. Natl. Acad. Sci. USA* **74**, 1736–1740 (1980).
8. T Veitshans, D Klimov, D Thirumalai, Protein folding kinetics: timescales, pathways and energy landscapes in terms of sequence-dependent properties. *Folding Des.* **2**, 1–22 (1997).
9. M Rubinstein, R Colby, *Polymer Physics.* (Oxford University Press Oxford, New York), (2003).
10. CJ Gommes, S Jaksch, H Frielinghaus, Small-angle scattering for beginners. *J. Appl. Crystallogr.* **54**, 1832–1843 (2021).
11. S Crick, M Jayaraman, C Frieden, R Wetzel, R Pappu, Fluorescence correlation spectroscopy shows that monomeric polyglutamine molecules form collapsed structures in aqueous solutions. *Proc. Natl. Acad. Sci. U. S. A.* **103**, 16764–16769 (2006).
12. R Walters, R Murphy, Examining polyglutamine peptide length: a connection between collapsed conformations and increased aggregation. *J. Mol. Biol* **393**, 978–992 (2009).

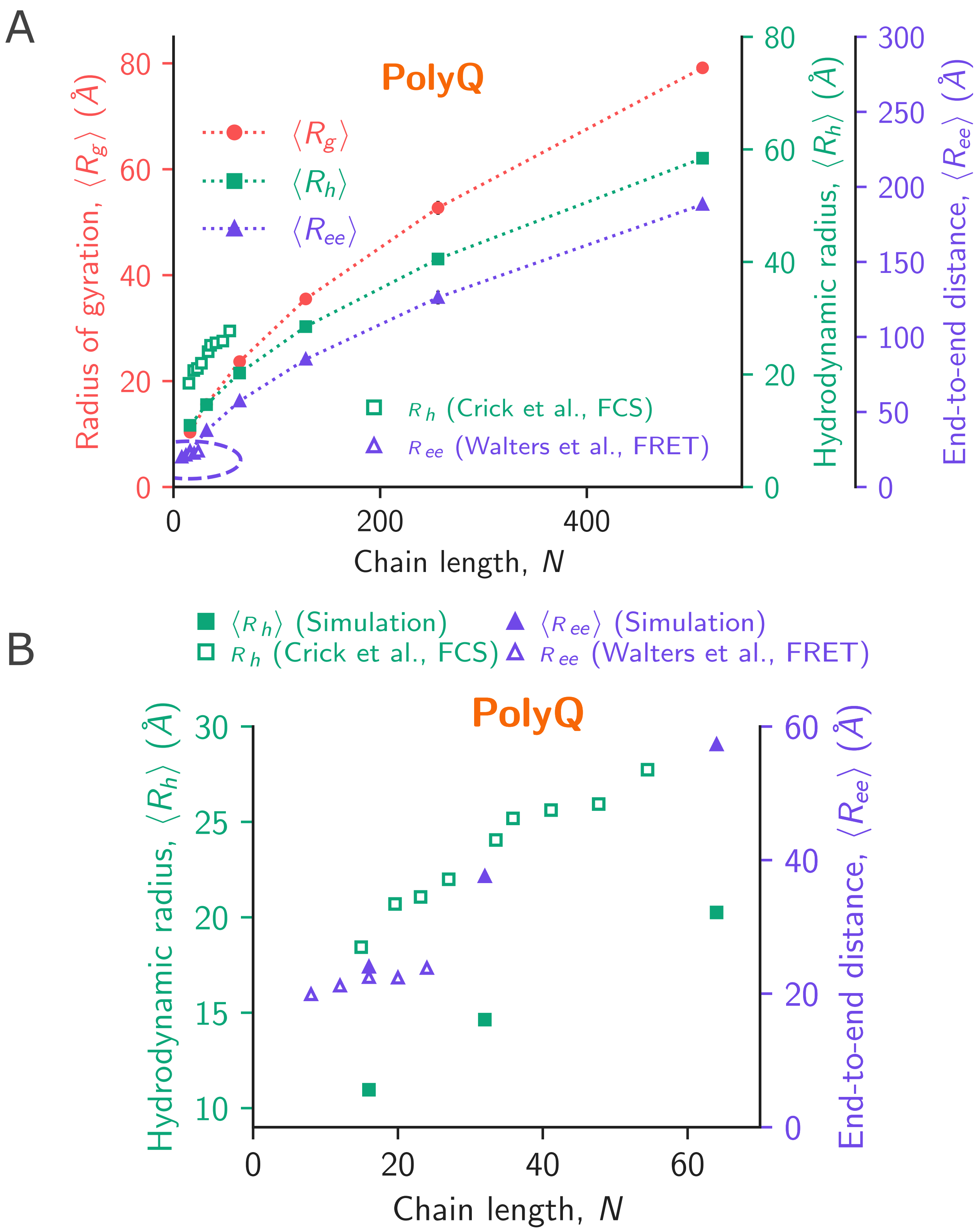

Fig. S1. (A) Dimensions of polyQ as a function of chain length N in terms of the radius of gyration $\langle R_g \rangle$ (red filled circles), hydrodynamic radius $\langle R_h \rangle$ (green filled squares) and end-to-end distance $\langle R_{ee} \rangle$ (violet filled triangles) in water (no cosolvent) computed from simulations. The experimental R_h (green open squares) and R_{ee} (violet open triangle) for polyQ chains are measured from FCS(11) and FRET(12) experiments, respectively. The FRET(12) data is highlighted with a violet dashed ellipse. (B) Average hydrodynamic radius, $\langle R_h \rangle$ (green squares) and end-to-end distance, $\langle R_{ee} \rangle$ (violet triangles) for polyQ are plotted as a function of chain length, N using the data from simulations (solid markers) and experiments (open markers). The experimental R_h and R_{ee} data are obtained from the FCS(11) and FRET(12) experiments. The error bars are of the marker size.

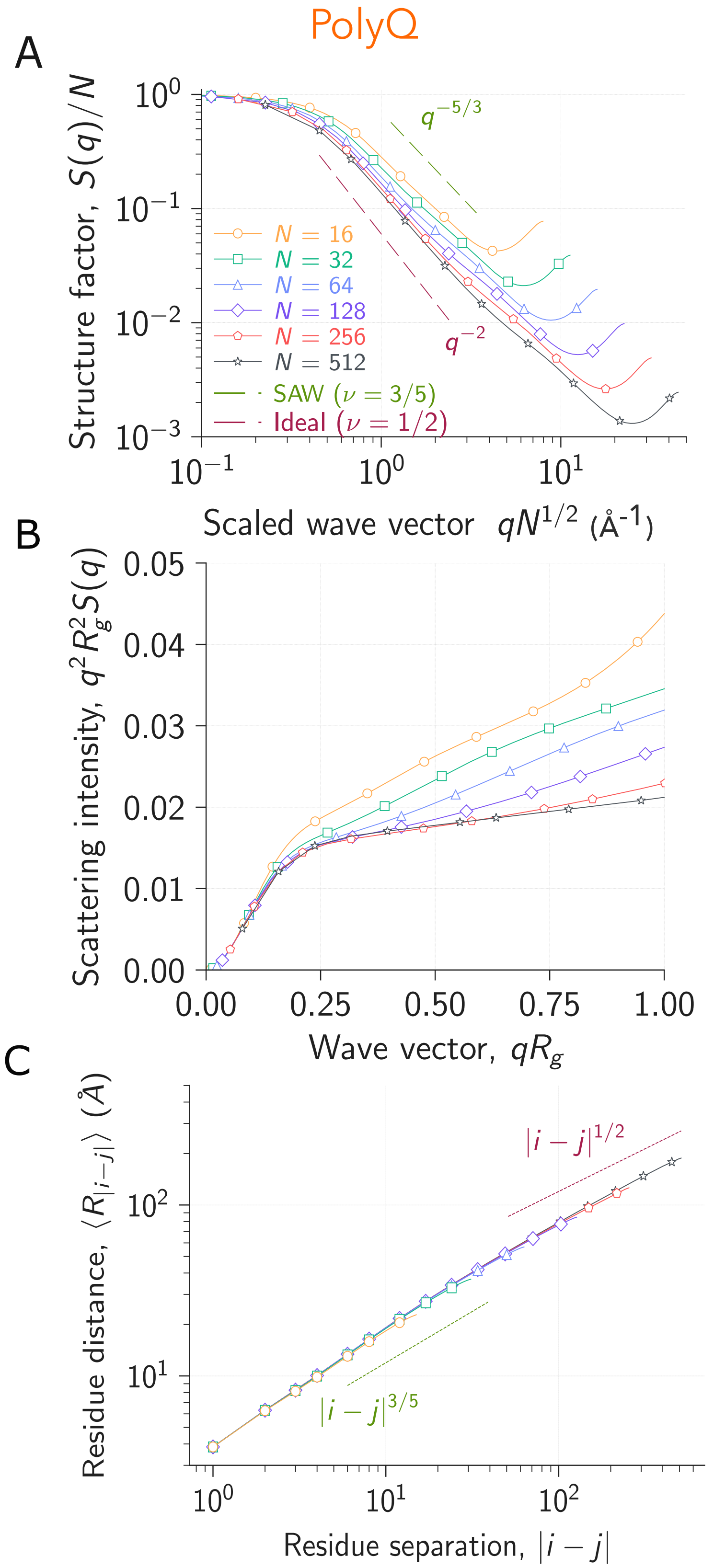

Fig. S2. Scaling behavior of polyQ with N in water (no cosolvent). (A) $S(q)/N$ plotted as a function of $qN^{1/2}$ for different N. (B) Dimensionless Kratky plots for different chain lengths. The slope of the plot at $qR_g \geq 0.2$ distinguishes the disordered (positive), Gaussian (zero) and folded/globule (negative) state of proteins. (C) $\langle R_{|i-j|}\rangle$ is plotted as a function of $|i-j|$. The annotations are the same for all the figures. The dashed lines in lime and purple show the scaling exponent ν = 3/5 and 1/2, respectively.

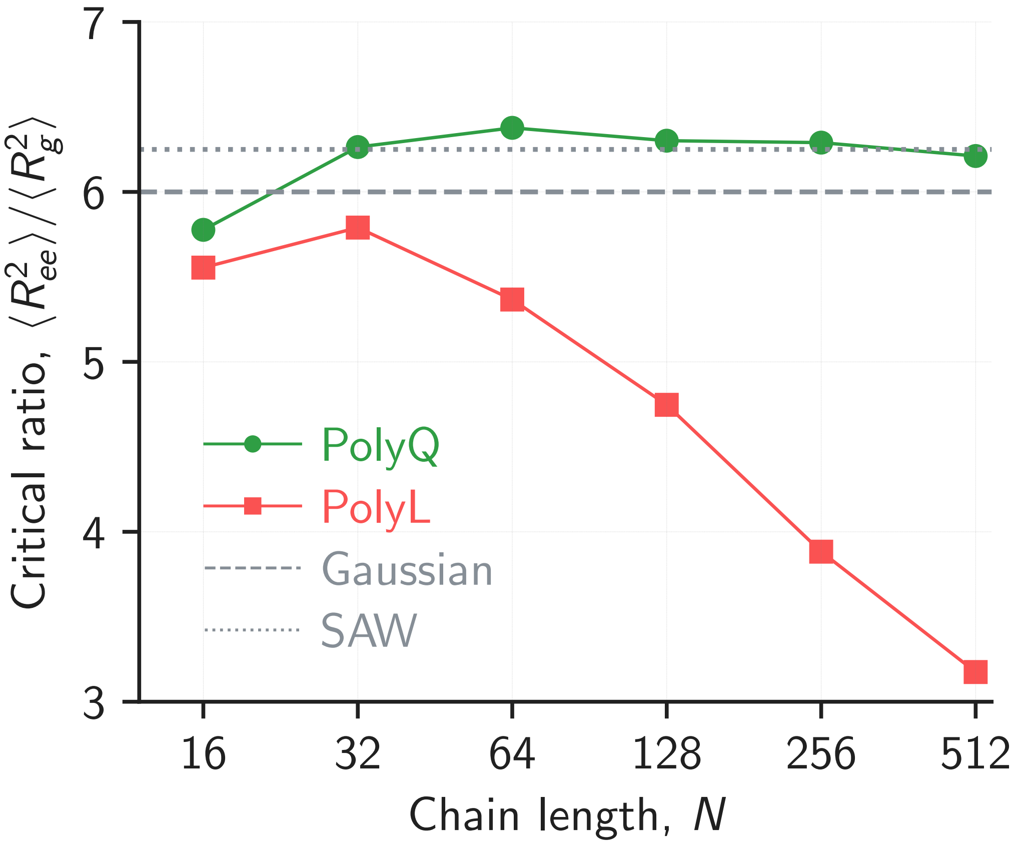

Fig. S3. Critical ratio $\langle R_{ee}^2 \rangle / \langle R_g^2 \rangle$ for polyQ (green circles) and polyL (red squares) in water are plotted as a function of chain length N. The grey dashed and dotted line corresponds to the critical ratio for the Gaussian ($\langle R_{ee}^2 \rangle / \langle R_g^2 \rangle$ = 6) and self-avoiding random walk polymer chain ($\langle R_{ee}^2 \rangle / \langle R_g^2 \rangle$ = 6.25).

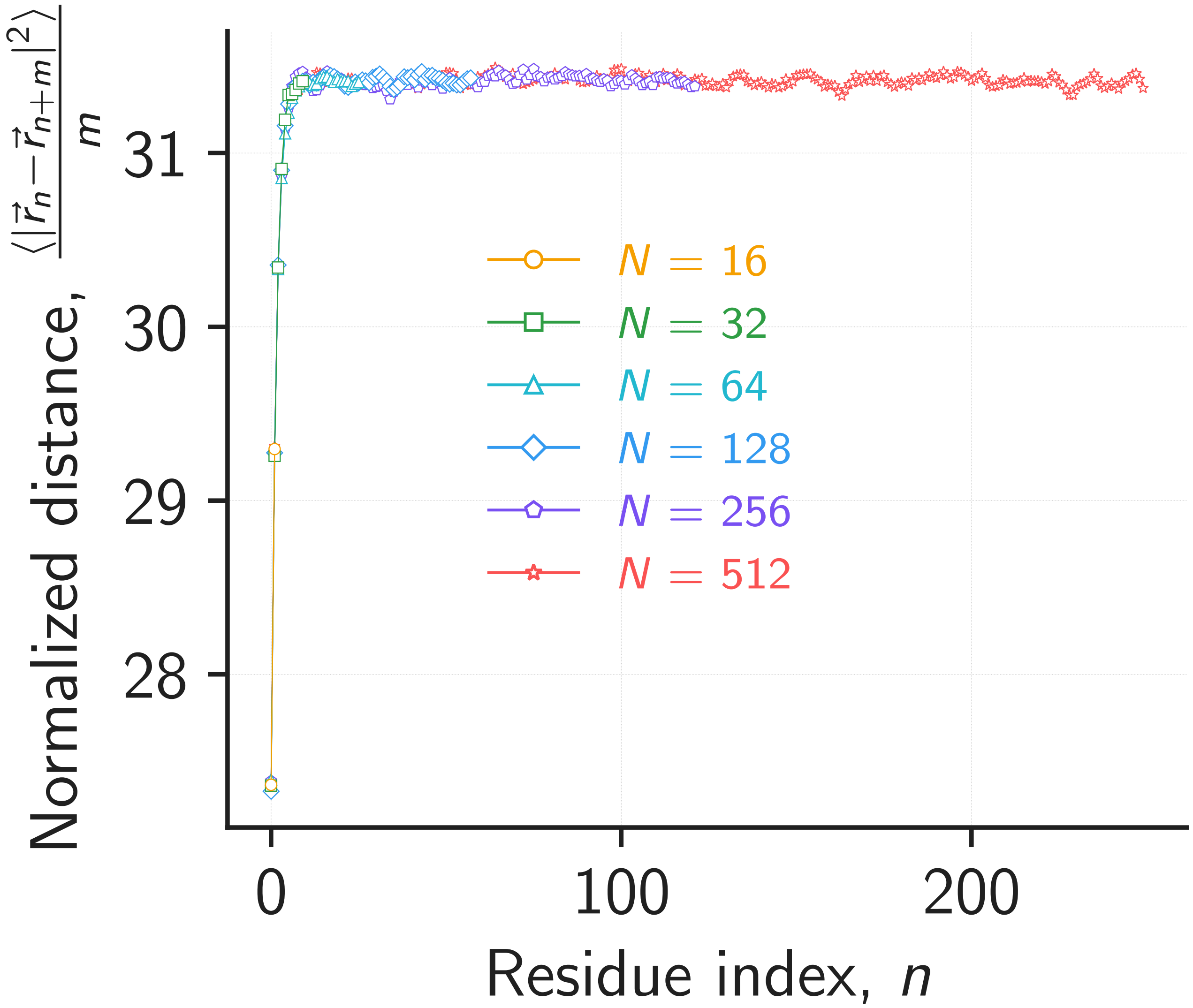

Fig. S4. Normalized mean-square distance between two backbone beads, n and $n+m$ along the chain as a function of n for polyQ chains with different N. We used m = 6 in the above plot.

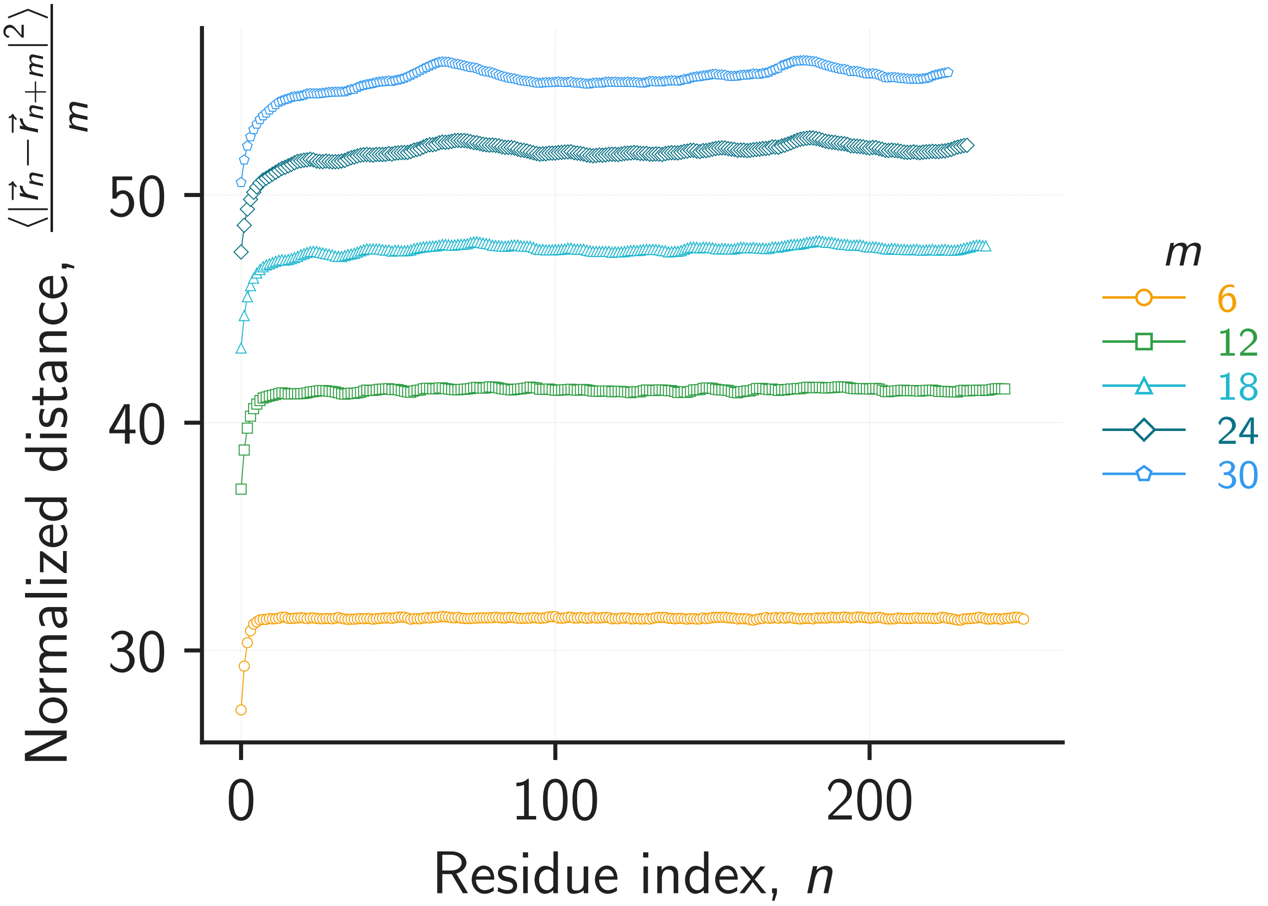

Fig. S5. Normalized mean-square distance between two backbone beads, n and $n+m$ along the chain as a function of n for polyQ with chain length N = 512. In the plot we varied m from 6 to 30.

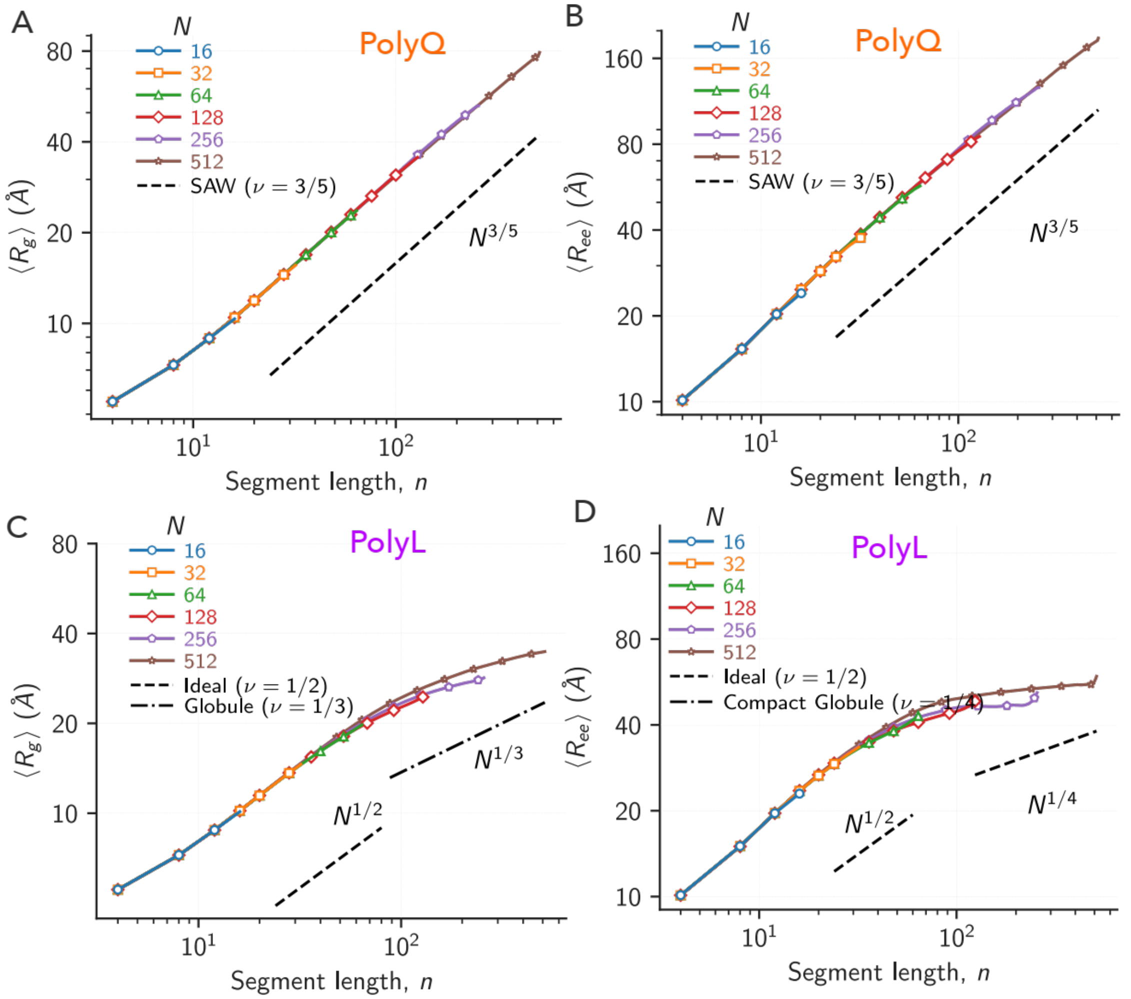

Fig. S6. (A) Radius of gyration, R_g and (B) end-to-end distance, R_{ee} as a function of chain segment length, n is plotted for polyQ for N = 16 to 512. Same for polyL in (C) and (D). The annotations for different chains are color-coded.

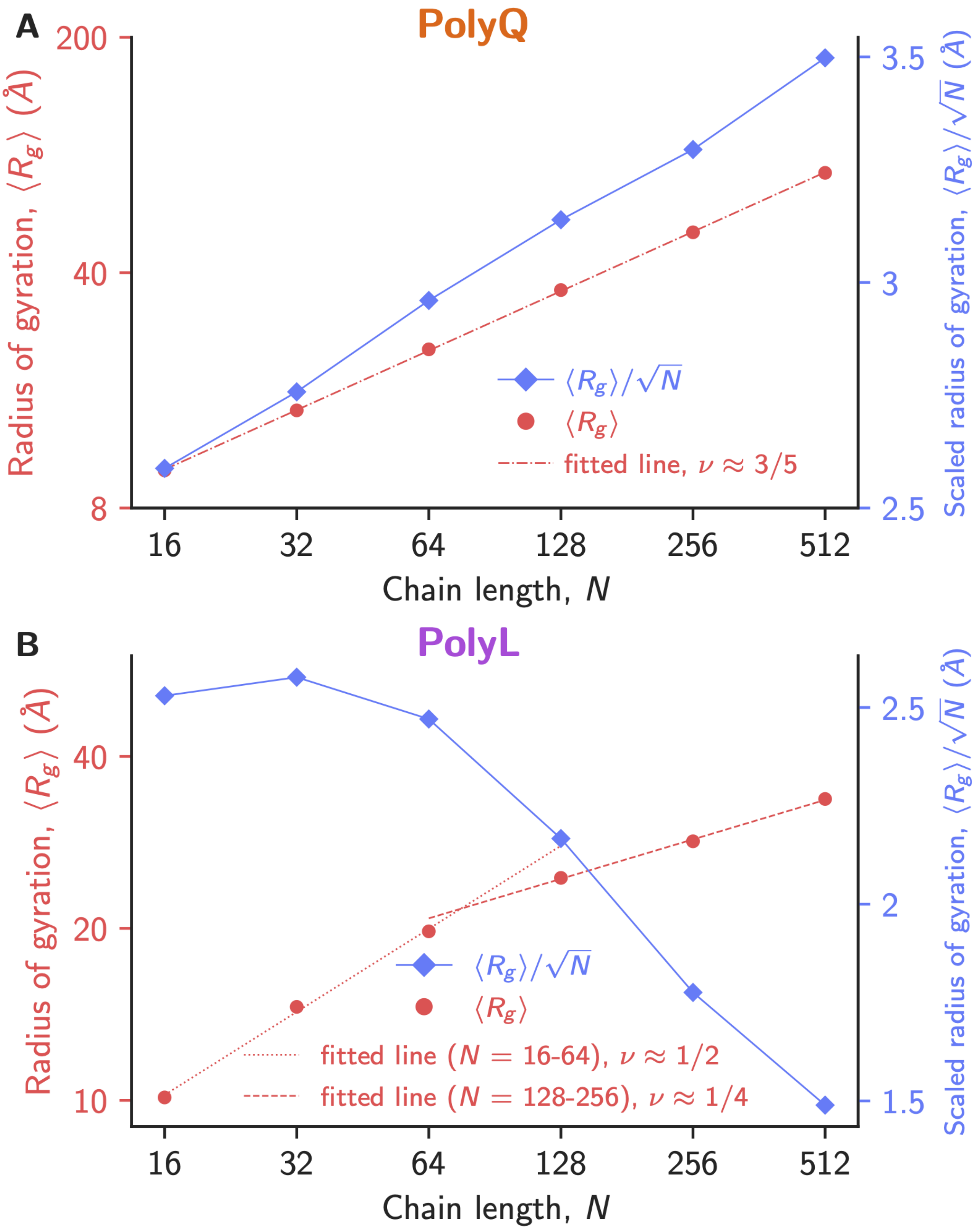

Fig. S7. Average radius of gyration, $\langle R_g \rangle$ (red circles) and scaled radius of gyration, $\langle R_g \rangle/\sqrt{N}$ (blue diamonds) as a function chain length, N for (A) polyQ and (B) polyL in water (no cosolvent). For polyQ chains (N = 16 - 512), the scaling exponent $\nu \approx 3/5$ is obtained from the fit of the $\langle R_g \rangle$ vs N (dashed-dotted line) plot. For polyL chains, $\nu \approx 1/2$ for smaller chain lengths (N = 16 - 64) and $\nu \approx 1/4$ for larger chain lengths (N = 128 - 512). The $\langle R_g \rangle$ error bars are of the marker size.

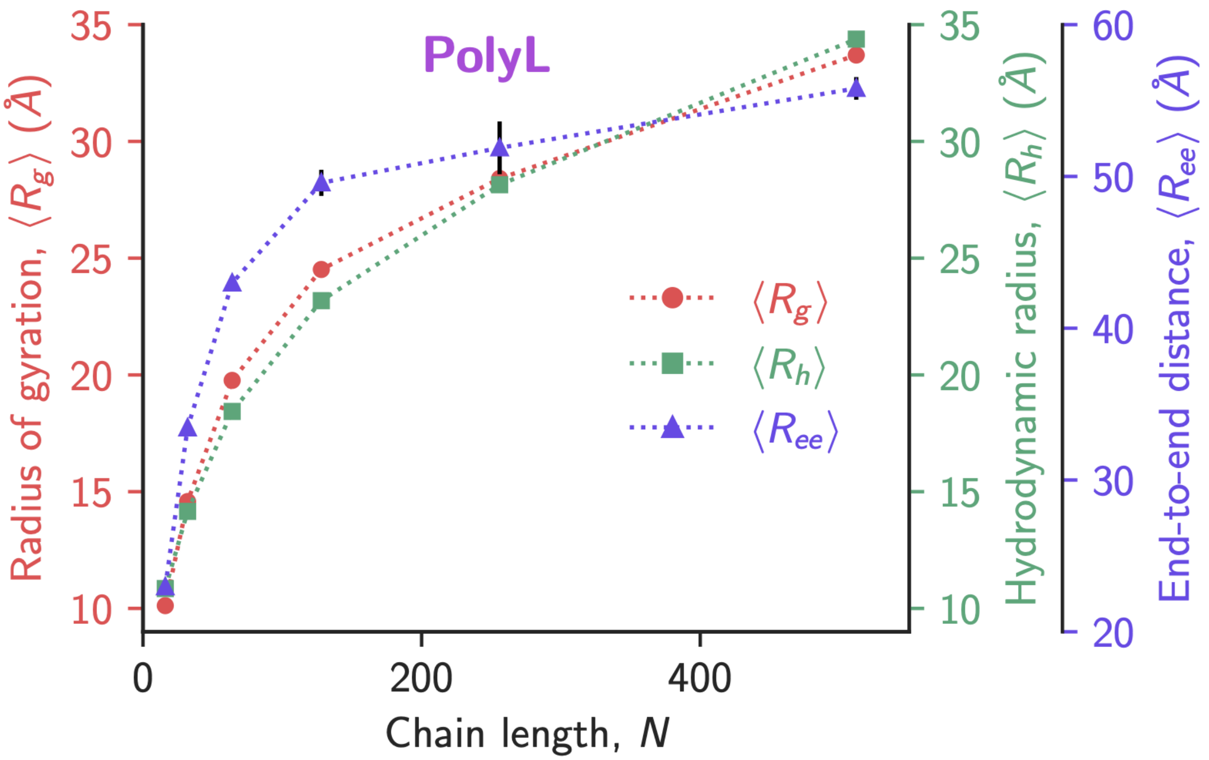

Fig. S8. Dimensions of polyL as a function of chain length N in terms of the radius of gyration $\langle R_g \rangle$ (red filled circles), hydrodynamic radius $\langle R_h \rangle$ (green filled squares) and end-to-end distance $\langle R_{ee} \rangle$ (violet filled triangles) in water (no cosolvent) computed from simulations. Error bars are shown with black vertical lines.

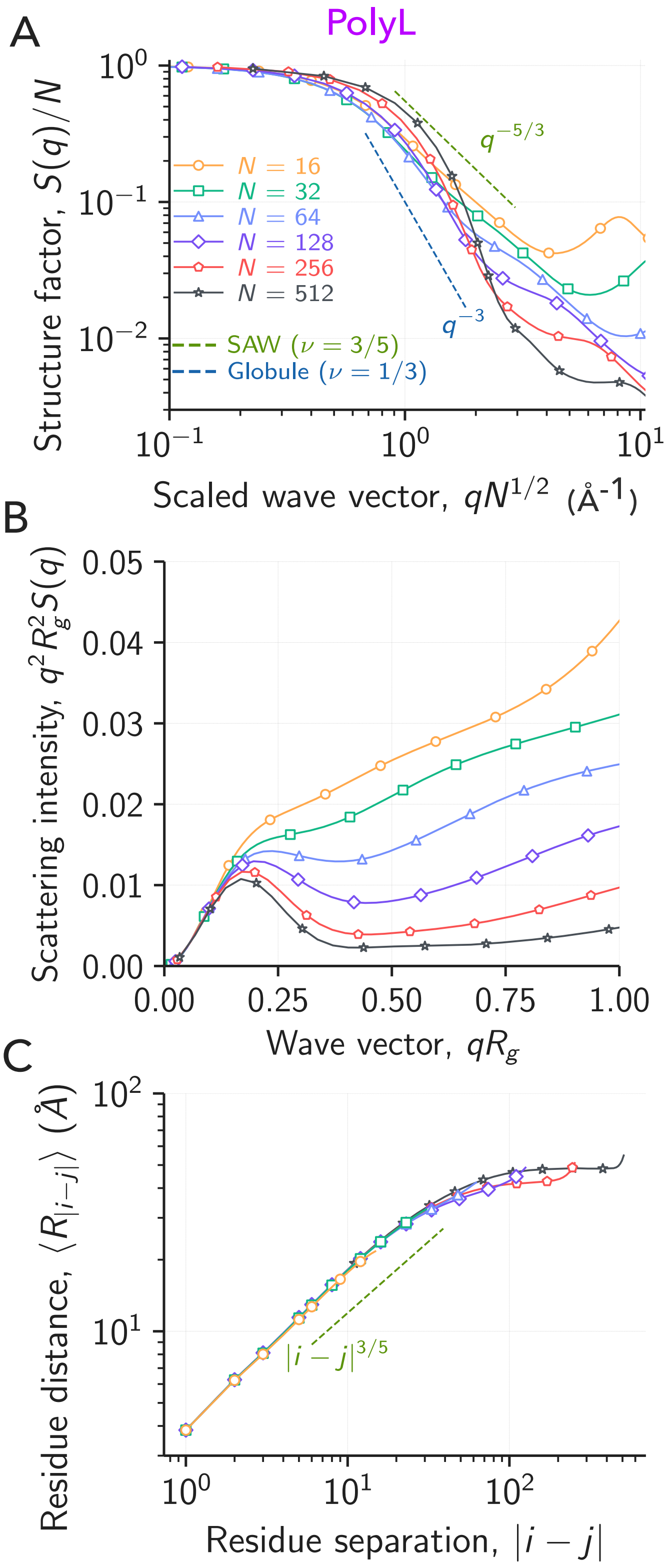

Fig. S9. Scaling behaviour of polyL with N in water (without cosolvent). (A) $S(q)/N$ as a function of $qN^{1/2}$ for different N. The dashed lines with scaling exponent ν = 3/5 and 1/3 correspond to a polymer exhibiting SAW (lime) and perfect globule state (blue) conformation. Steeper slopes are signatures of Porod Scattering. (B) Dimensionless Kratky plots for different chain lengths. The slope of the plot at $qR_g \geq 0.2$ distinguishes the disordered (positive), Gaussian (zero) and folded/globule (negative) state of proteins. (C) $\langle R_{|i-j|}\rangle$ is plotted as a function of $|i-j|$. The annotations are the same for all the figures.

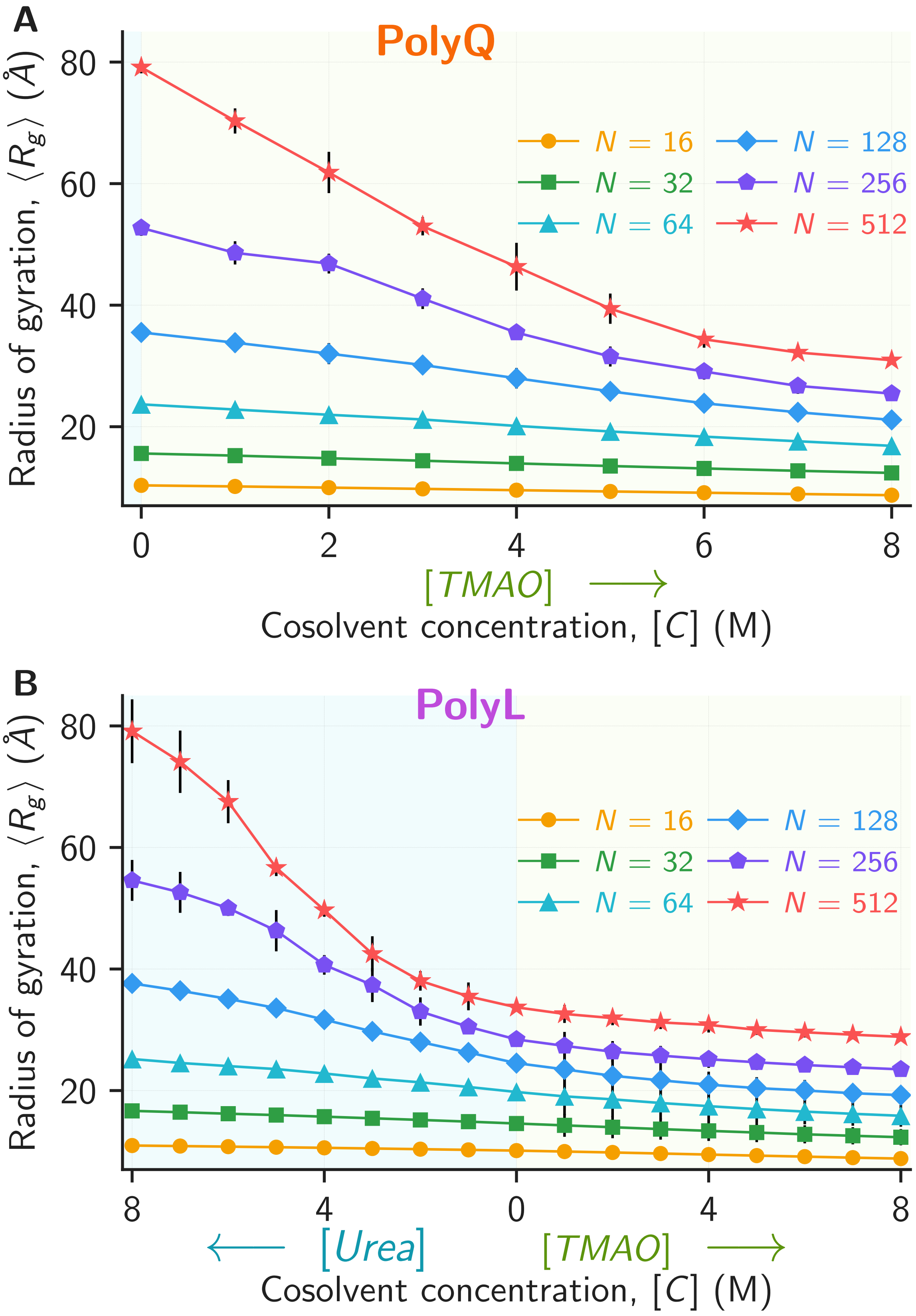

Fig. S10. $\langle R_g \rangle$ plotted as a function of cosolvent concentration, $[C]$ for (A) polyQ and (B) polyL. The chain lengths are mentioned in the annotation. The shaded regions correspond to cosolvent TMAO (lime) and urea (cyan).

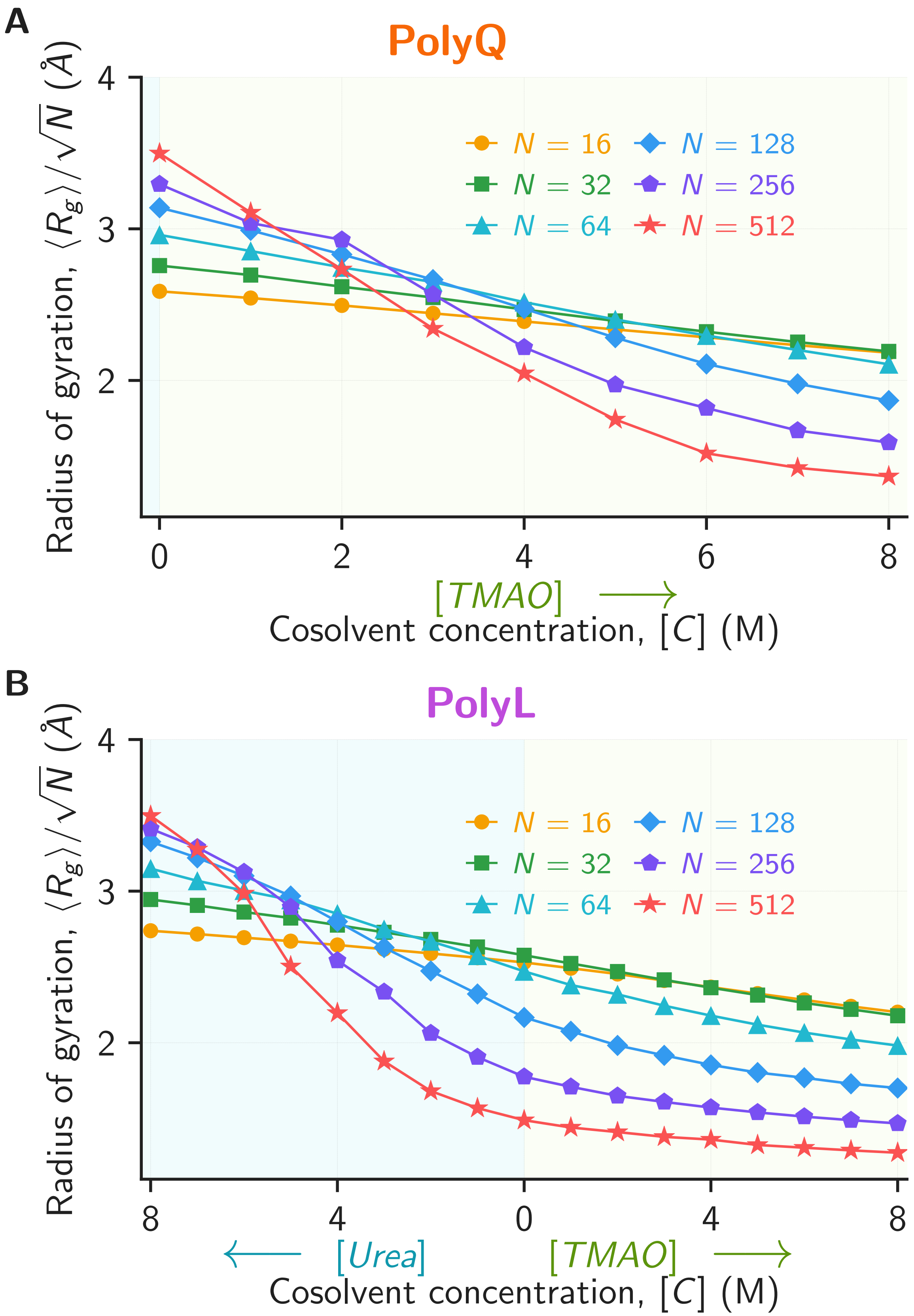

Fig. S11. Normalized radius of gyration, $\langle R_g \rangle / \sqrt{N}$ as a function of cosolvent concentration, $[C]$ for (A) polyQ and (B) polyL. The chain lengths are mentioned in the annotation. The shaded regions correspond to cosolvent TMAO (lime) and urea (cyan).

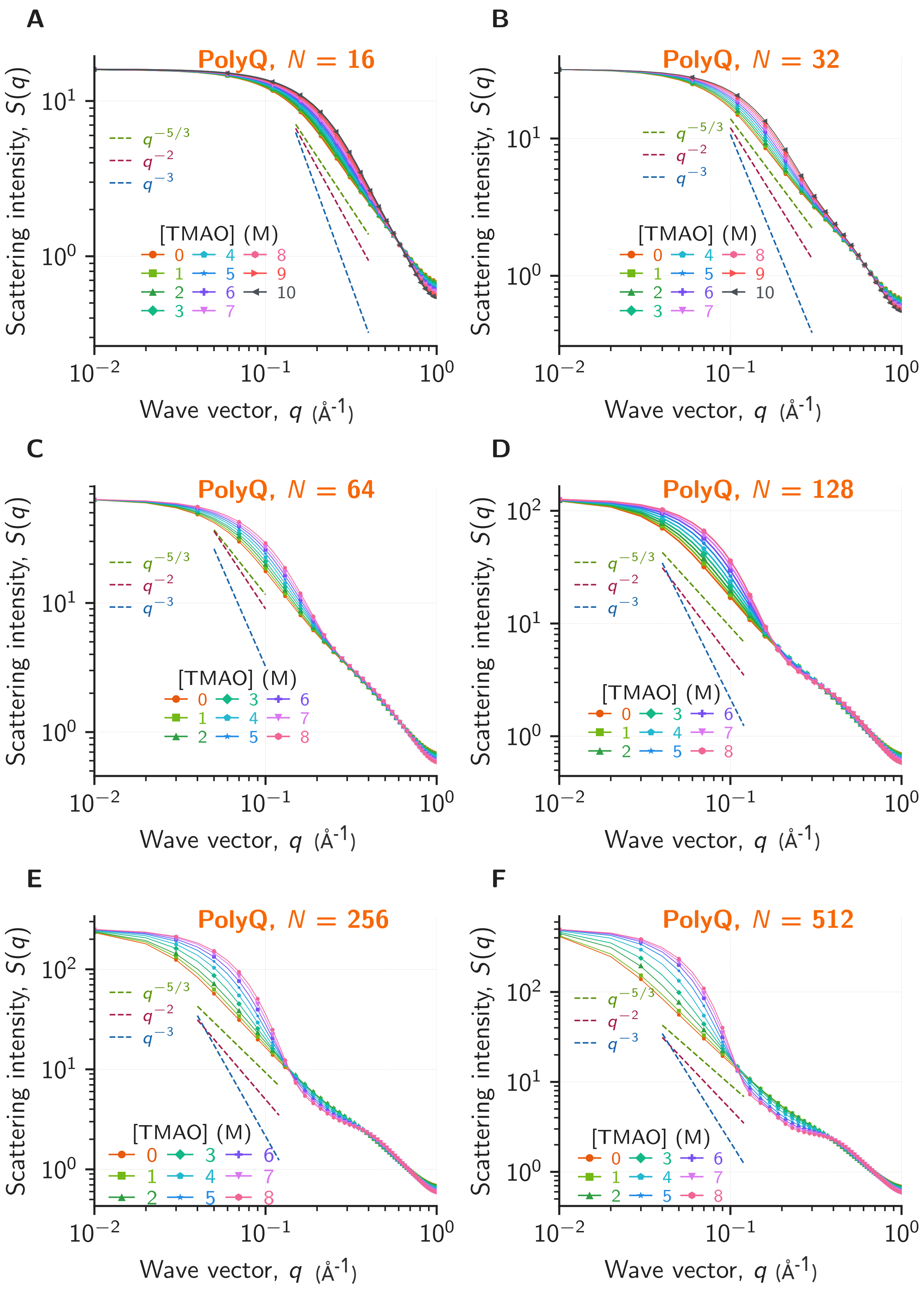

Fig. S12. Scattering function $S(q)$ of polyQ plotted as a function of wave vector q in various TMAO concentrations for (A) $N = 16$, (B) $N = 32$, (C) $N = 64$, (D) $N = 128$, (E) $N = 256$ and (F) $N = 512$. The different cosolvent concentrations are mentioned in the annotation.

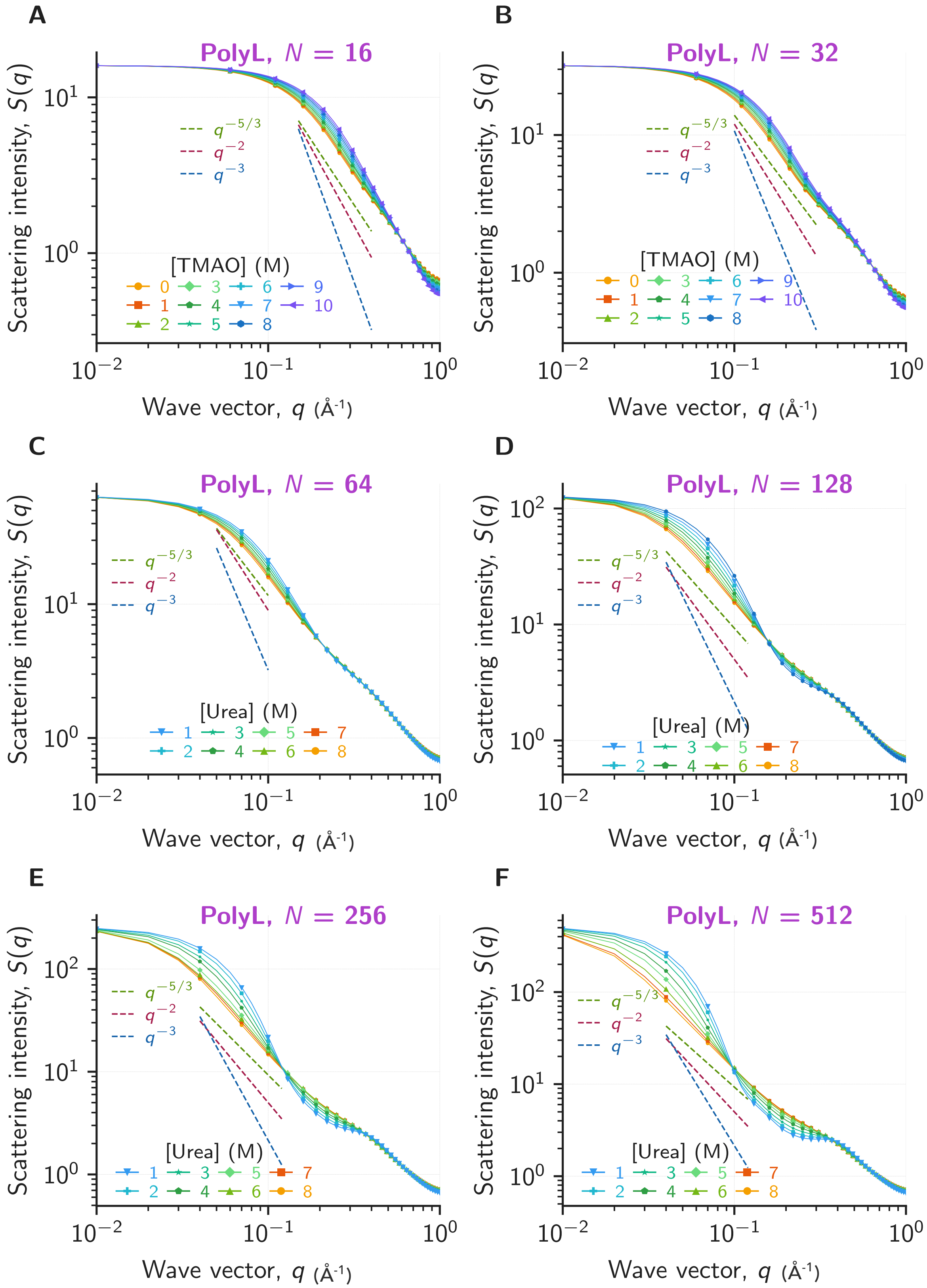

Fig. S13. Scattering function $S(q)$ of polyL plotted as a function of wave vector q in various cosolvent concentrations for (A) $N = 16$, (B) $N = 32$, (C) $N = 64$, (D) $N = 128$, (E) $N = 256$ and (F) $N = 512$. The different cosolvent concentrations are mentioned in the annotation.

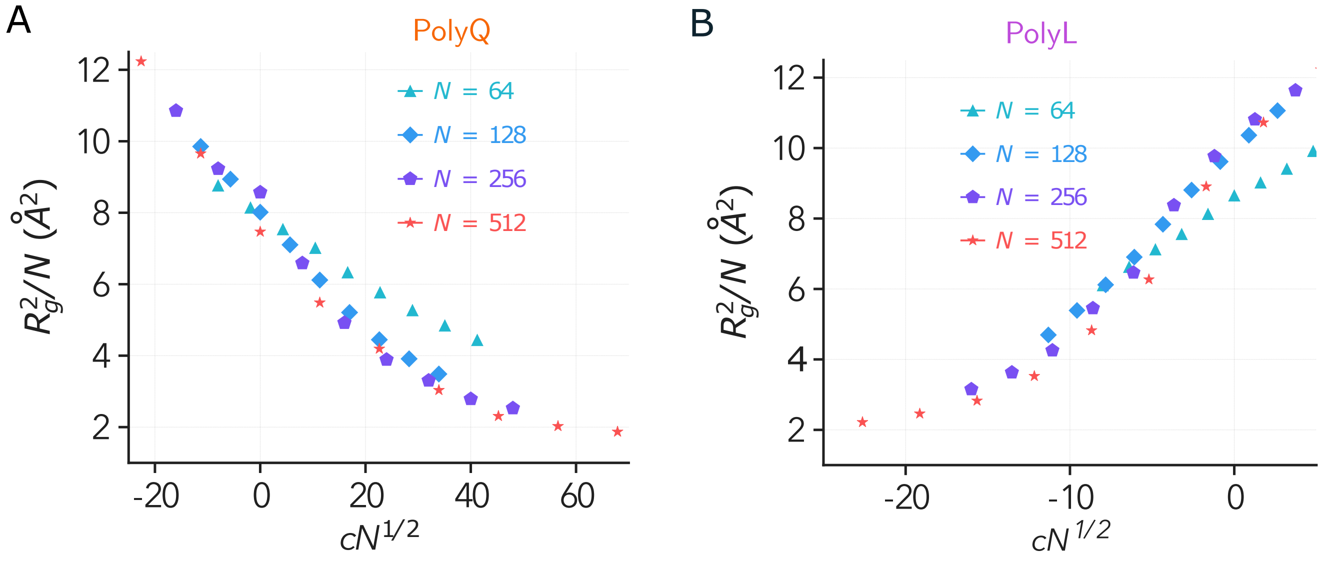

Fig. S14. R_g^2/N is plotted as a function of $cN^{1/2}$ for (A) polyQ and (B) polyL at various cosolvent concentrations, where $c = ([C]/[C_\Theta] - 1)$. For polyQ and polyL, the cosolvent concentration at Θ point, C_Θ are $[TMAO]$ = 2 M and $[urea]$ = 6.49 M, respectively. The data for N = 64 deviates from the scaling relation.

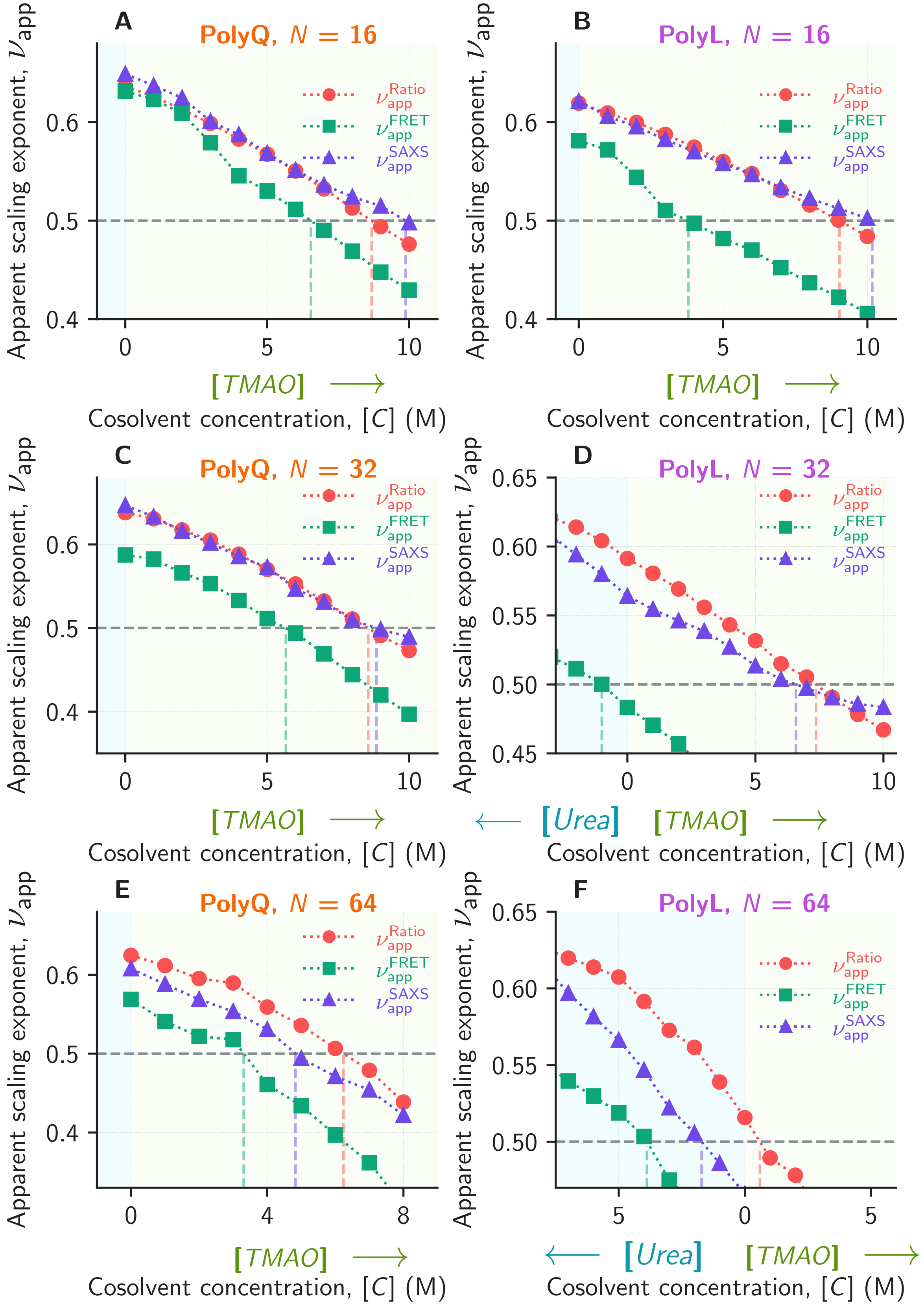

Fig. S15. The apparent scaling exponent ν_{app} as a function of cosolvent concentration $[C]$ extracted using three methods - critical ratio (red circles), pair distances mimicking FRET (green squares) and structure factor mimicking SAXS (violet triangles) for (A) polyQ, N = 16, (B) polyL, N = 16, (C) polyQ, N = 32, (D) polyL, N = 32, (E) polyQ, N = 64 and (F) polyL, N = 64. The grey horizontal dashed line denotes the CG transition (Θ-region) where ν_{app} = 1/2. The three vertical dashed lines correspond to the coil-globule transition concentration $[C_{\Theta,N}]$ from three different methods. The green and cyan shaded regions correspond to the cosolvents, TMAO and urea, respectively.

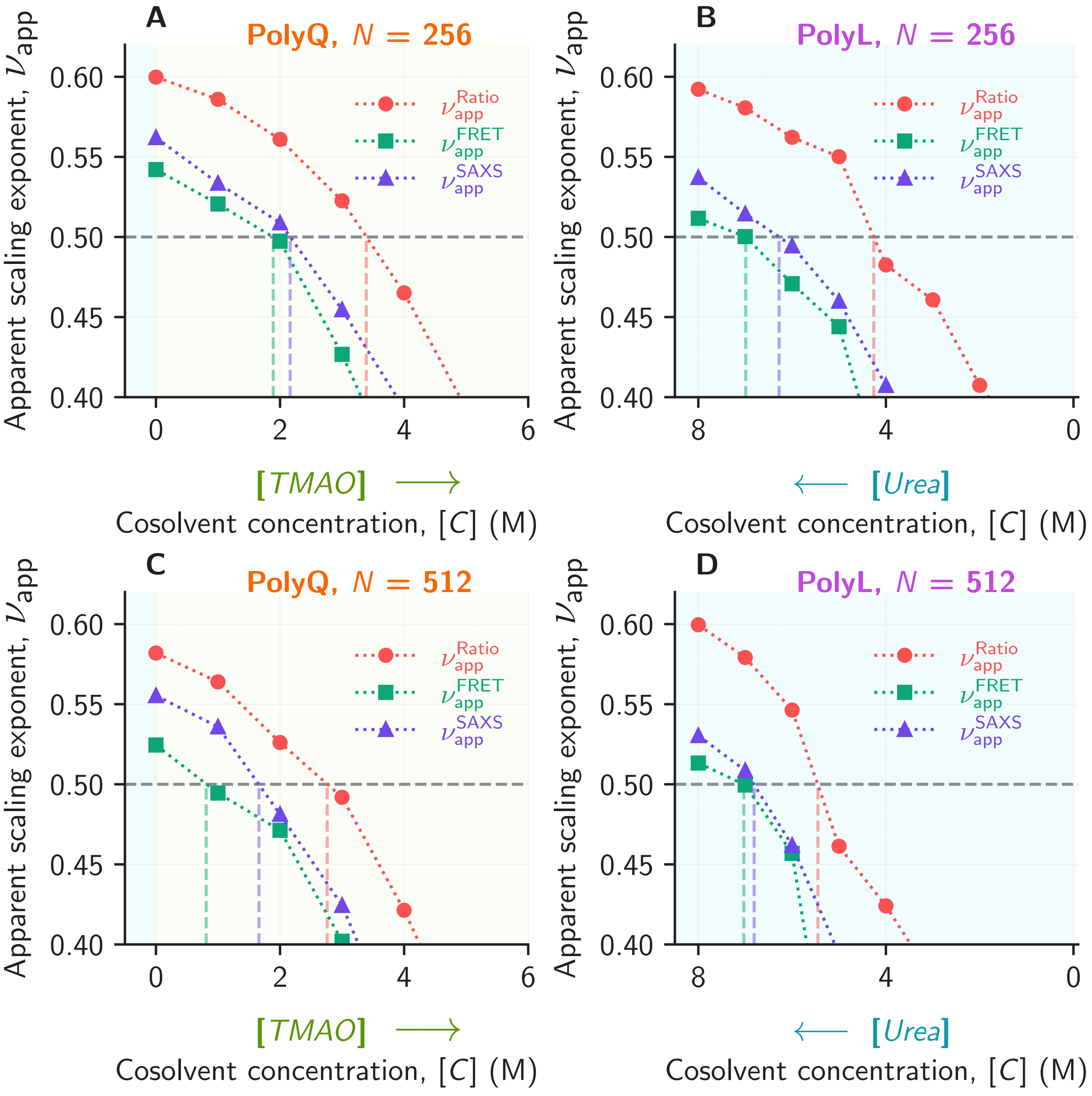

Fig. S16. The apparent scaling exponent ν_{app} as a function of cosolvent concentration $[C]$ extracted using three methods - critical ratio (red circles), pair distances mimicking FRET (green squares) and structure factor mimicking SAXS (violet triangles) for (A) polyQ, N = 256 (B) polyL, N = 256, (C) polyQ, N = 512 and (D) polyL, N = 512. The grey horizontal dashed line denotes the CG transition (Θ-region) where ν_{app} = 1/2. The three vertical dashed lines correspond to the coil-globule transition concentration $[C_{\Theta,N}]$ from three different methods. The green and cyan shaded regions correspond to the cosolvents, TMAO and urea, respectively.

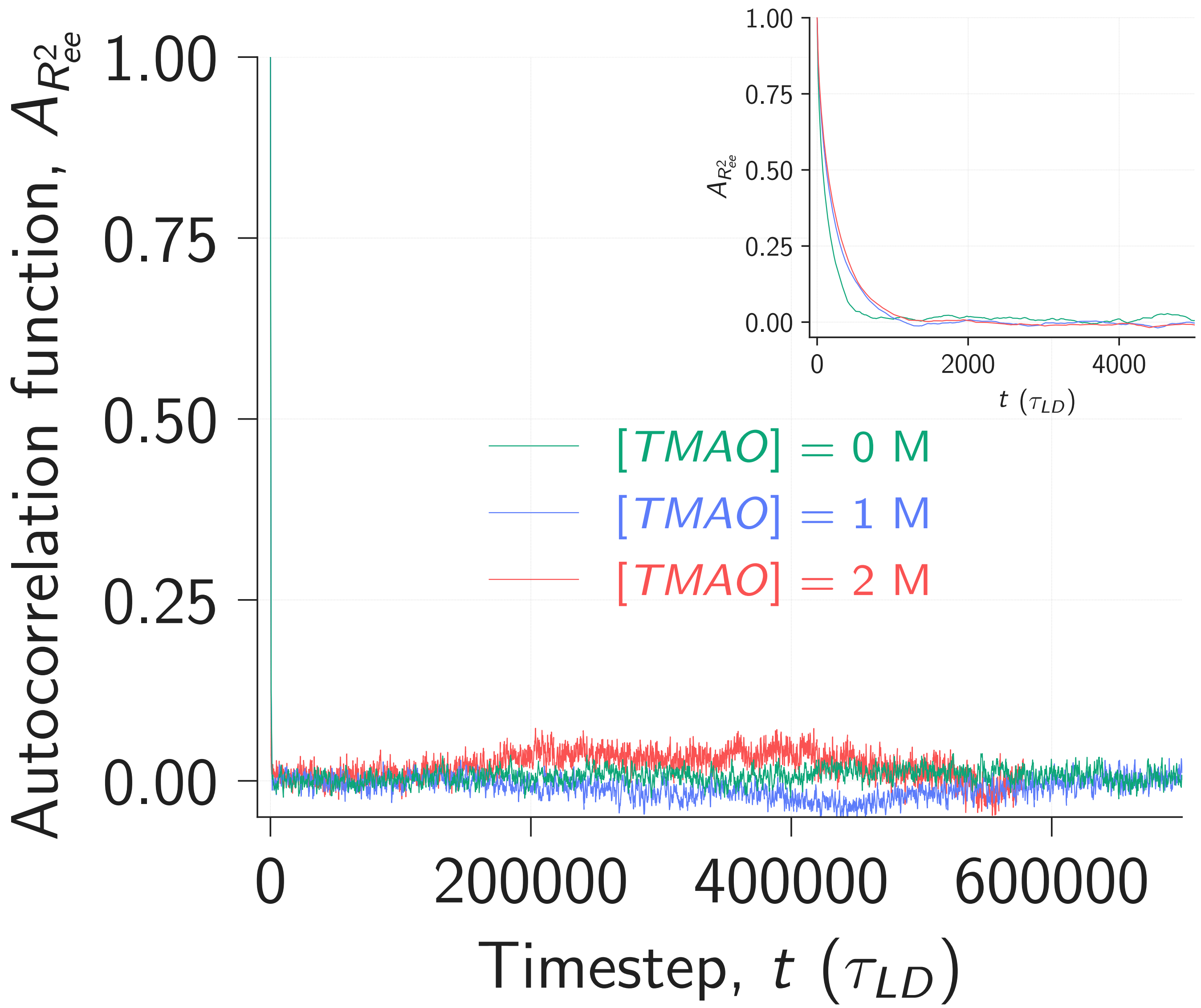

Fig. S17. End-to-end distance autocorrelation function, $A_{R^2_{ee}}$ as a function of timestep, t for polyQ, N = 512. Smooth decay of autocorrelation function is shown in inset.

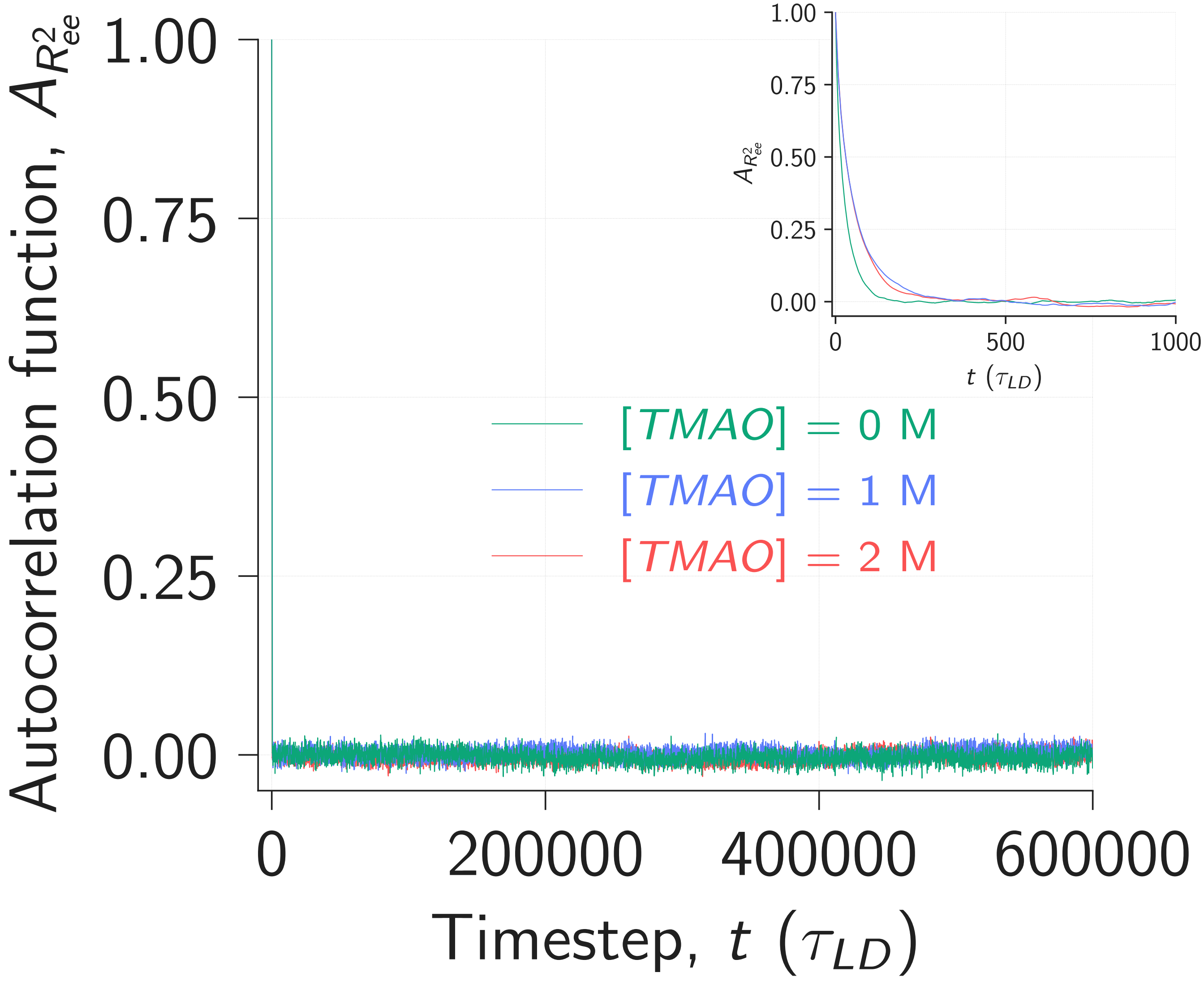

Fig. S18. End-to-end distance autocorrelation function, $A_{R^2_{ee}}$ as a function of timestep, t for polyQ, N = 64. Smooth decay of autocorrelation function is shown in inset.

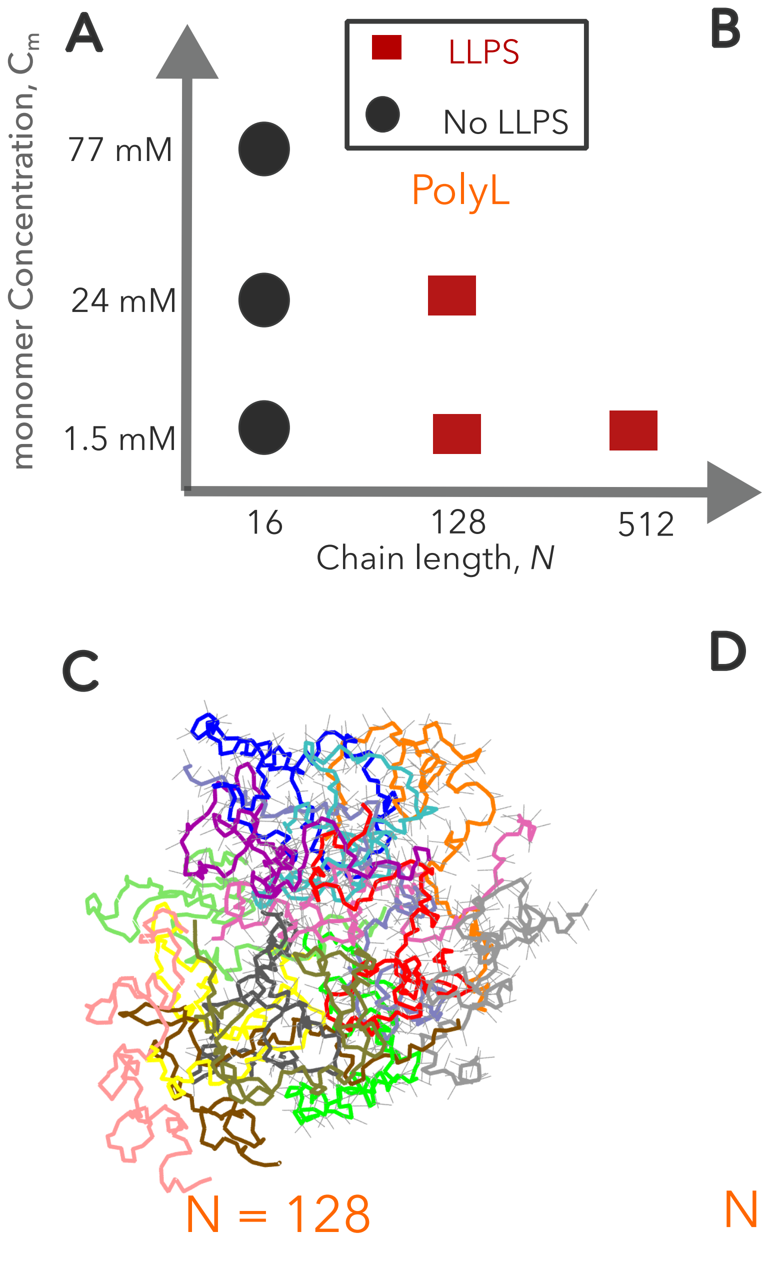

Fig. S19. (A) Droplet formation for different monomer concentration, C_m (= $N * N_{ch}/V$ where N, N_{ch} and V are the chain length, number of chains, volume of the box) for various chain length, N of polyL. Red (grey) square denotes presence (absence) of droplet. Representative snapshots from multichain simulations for polyL chains, (B) N = 16, (C) N = 128 and (D) N = 512.
